# Fibroadipogenic progenitors mediate the ability of HDAC inhibitors to promote regeneration in dystrophic muscles of young, but not old Mdx mice

**DOI:** 10.1002/emmm.201202096

**Published:** 2013-03-18

**Authors:** Chiara Mozzetta, Silvia Consalvi, Valentina Saccone, Matthew Tierney, Adamo Diamantini, Kathryn J Mitchell, Giovanna Marazzi, Giovanna Borsellino, Luca Battistini, David Sassoon, Alessandra Sacco, Pier Lorenzo Puri

**Affiliations:** 1 IRCCS Fondazione Santa LuciaRome, Italy; 2 Sanford-Burnham Medical Research Institute, Sanford Children's Health Research CenterLa Jolla, CA, USA; 3 Myology Group, UMR S 787 INSERM, Université Pierre et Marie Curie Paris VIParis, France

**Keywords:** fibroadipogenic progenitors, HDAC inhibitors, muscle regeneration, muscle stem cells, muscular dystrophy

## Abstract

HDAC inhibitors (HDACi) exert beneficial effects in mdx mice, by promoting endogenous regeneration; however, the cellular determinants of HDACi activity on dystrophic muscles have not been determined. We show that fibroadipogenic progenitors (FAP) influence the regeneration potential of satellite cells during disease progression in mdx mice and mediate HDACi ability to selectively promote regeneration at early stages of disease. FAPs from young mdx mice promote, while FAPs from old mdx mice repress, satellite cell-mediated formation of myotubes. In young mdx mice HDACi inhibited FAP adipogenic potential, while enhancing their ability to promote differentiation of adjacent satellite cells, through upregulation of the soluble factor follistatin. By contrast, FAPs from old mdx mice were resistant to HDACi-mediated inhibition of adipogenesis and constitutively repressed satellite cell-mediated formation of myotubes. We show that transplantation of FAPs from regenerating young muscles restored HDACi ability to increase myofibre size in old mdx mice. These results reveal that FAPs are key cellular determinants of disease progression in mdx mice and mediate a previously unappreciated stage-specific beneficial effect of HDACi in dystrophic muscles.

## INTRODUCTION

Pharmacological control of tissue and organ regeneration is a suitable strategy to counter the progression of neuromuscular degenerative diseases for which there is currently no available therapy. Muscular dystrophies (MDs) include hereditary, fatal disorders characterized by the progressive, functional decline of skeletal muscles, caused by mutations of genes implicated in the maintenance of myofibre integrity (Dalkilic & Kunkel, 2003). A key feature of the natural history in most MDs is the initial compensatory response of degenerating muscles through a reactive regeneration that tends to counterbalance muscle loss (Mozzetta et al, 2009). However, as the disease progresses, the regenerative potential of dystrophic muscles declines and myofibre repair becomes biased toward the formation of fibrotic scars and fat infiltration (Serrano et al, 2011). Thus, interventions that simultaneously promote muscle regeneration and prevent fibro-adipogenic degeneration provide the most desirable treatment for MDs. *In situ* reprogramming of adult pluripotent cell types that contribute to muscle regeneration by epigenetic drugs is a suitable approach in the pharmacological treatment of MDs. However, its application is hampered by the current paucity of information on the identity of the cellular target(s) and the relative impact of environmental cues in directing cell reprogramming toward specific lineages to promote therapeutic effects. We have previously reported on the ability of histone deacetylase inhibitors (HDACi) to promote functional and morphological recovery of dystrophic muscles, by enhancing endogenous regeneration and increasing the myofibre size, while preventing fibrotic scars and fat deposition (Minetti et al, 2006). Yet, the cellular and molecular effectors of such beneficial effect remain unknown. Although muscle satellite cells (MuSCs) are the principal contributors to the regeneration of injured and diseased muscles, it is becoming apparent that their activity is influenced by environmental cues derived from the inflammatory infiltrate and other cell types (Brack & Rando, 2007; Kuang & Rudnicki, 2008; Shi & Garry, 2006). In particular, reciprocal, functional interactions between distinct cell populations present in injured or diseased muscles appear to determine whether repair occurs by either regeneration or fibro-adipogenic degeneration. For instance, while skeletal muscles at early stages of MD are permissive for the satellite cell-mediated regeneration, late stages of disease correlate with formation of fibrotic scars and fat deposition that bias the environment toward the inhibition of satellite cell activity.

The recent identification of muscle-derived interstitial cells that can adopt multiple lineages and contribute, either directly or indirectly, to muscle regeneration (Joe et al, 2010; Mitchell et al, 2010; Rodeheffer, 2010; Uezumi et al, 2010) indicates a previously unrecognized complexity in the regulation of muscle homeostasis and regeneration. These cells probably belong to a heterogeneous population of intramuscular, multipotent cells displaying overlapping cell surface markers, such as Sca1 (Natarajan et al, 2010). Sca1 positive (Sca1^pos^) resident muscle interstitial cells have been reported to contribute to muscle regeneration or fibrosis by previous works (Hidestrand et al, 2008; Kafadar et al, 2009; Mitchell et al, 2005, 2010). Two recent reports have described the identification of muscle-derived interstitial cells that were sorted based on their high Sca1 expression (Joe et al, 2010) or PDGF receptor alpha (PDGF-R-alpha) expression (Uezumi et al, 2010), respectively. Interestingly, these cell populations share similar biological properties, such as the ability to turn into fibro-adipocytes in response to signals released by degenerating muscles. Since ectopic fat formation and fibrotic scars are detrimental common outcomes of degenerative muscle disorders, these cells are interesting candidates as cellular determinants of disease progression. An additional biological property of one of these cell populations – collectively referred to as fibro-adipocyte progenitors (FAPs; Joe et al, 2010) – relates to their reciprocal interactions with myofibres and satellite cells. In resting muscles, the interaction with intact myofibres prevents their conversion into fibro-adipocytes (Uezumi et al, 2010); however, muscle injury stimulates these cells to produce paracrine factors that promote satellite cell-mediated regeneration (Joe et al, 2010). By contrast, in degenerating muscles, such as dystrophic muscles at advanced stages of disease, these cells turn into fibro-adipocytes, which mediate fat deposition and fibrosis (Uezumi et al, 2011), thereby disrupting the environment conducive for muscle regeneration. Thus, these cells might contribute to the pathogenesis of MDs indirectly, by influencing the activity of satellite cells, and directly by promoting fibroadipogenic degeneration. Therefore they provide a valuable target for interventions toward shifting the balance between muscle regeneration and fibroadipogenic degeneration in MDs, such as in the case of pharmacological blockade of HDAC (Consalvi et al, 2011).

## RESULTS

### Functional exhaustion of regeneration in dystrophic muscles at late stage of disease progression coincides with an impaired ability of FAPs to support MuSC myogenic potential

To determine the relative contribution of MuSCs and FAPs to the exhaustion of muscle regeneration occurring at late stages of MD, we isolated these two cellular populations from muscles of 1.5-month-old wild type (wt) mice or from muscles of mdx mice at different stages of disease progression – 1.5 months (young mdx) and 12 months (old mdx). Supporting Information Fig S1 shows that FAPs and MuSCs can be isolated by FACS as two clearly distinct populations, based on the differential expression of surface markers. According to previous works, MuSCs were isolated as α7-integrin^pos^/Sca1^neg^ cells (Sacco et al, 2008), while FAPs were isolated as α7integrin^neg^/Sca1^pos^ cells (Joe et al, 2010). Supporting Information Fig S1 shows comparable FACS profiles of MuSCs and FAPs isolated from muscles of wild type or mdx mice, regardless the age and stage of disease, although a slight, but reproducible decrease in the amount of satellite cells was observed in old mdx mice.

**Figure 1:**
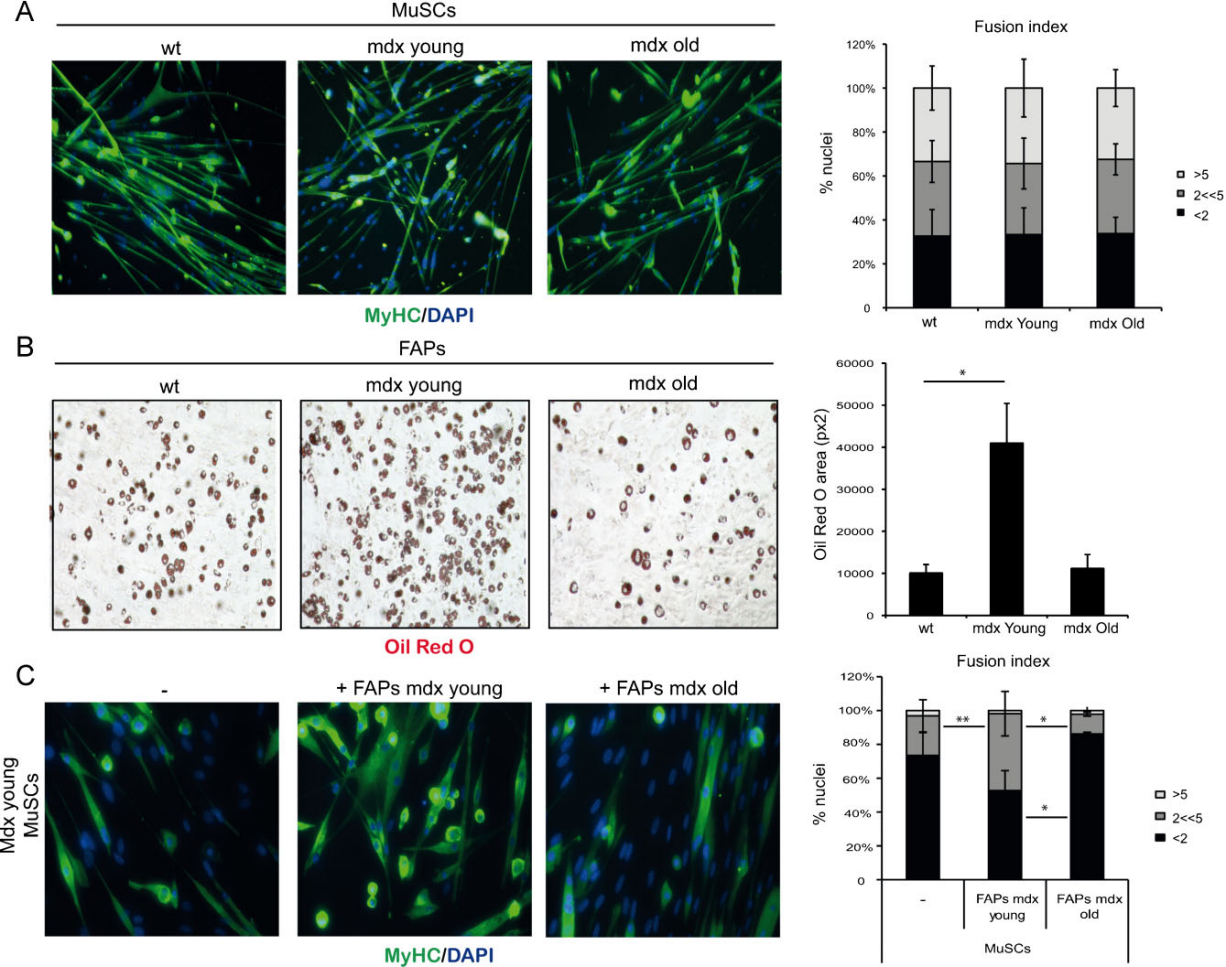
Intrinsic differentiation potential and functional interactions between MuSCs and FAPs in MDX mice at different stages of disease. A. MuSCs isolated from hindlimbs muscles of wild type (wt) mice or mdx mice at early (mdx Young, 1.5-month old) or late (mdx Old, 12-month old) stages of disease progression (*n* = 3). Left panel: the myogenic potential of MuSCs was assessed by MyHC immunofluorescence. Representative images of MyHC (green) staining are shown. Nuclei were counterstained with DAPI (blue). Right panel: graph showing the quantification of fusion index. We measured the percentage of nuclei that were MyHC^−^ or MyHC^+^ in mononucleated myotubes (<2), the % of nuclei that were inside myotubes containing between 2 and 5 nuclei (2 << 5) and the % of nuclei inside myotubes containing more than five nuclei (>5). Data are represented as average ± SEM. B. FAPs were isolated from hindlimbs muscles of wild type (wt) mice or mdx mice at early (mdx Young, 1.5 month old) or late (mdx Old, 12 month old) stages of disease progression (*n* ≥ 3). Left panel: the adipogenic potential of FAPs was measured by Oil red O staining after 7 days of culture in growth medium and subsequent 6 days culture in adipogenic differentiation medium (adp-DM). Right panel: graph showing the quantification of red oil area, reported as pixel^2^/field. Data are represented as average ± SEM; statistical significance assessed by *t*-test, *p < 0.05. C. Left panel: The ability of FAPs, isolated from either young (middle panel) or old (right panel) mdx mice, to enhance MuSC ability to form myotubes was determined by MyHC staining after 7 days of co-culture in growth medium. Right panel: graph showing the quantification of fusion index. We measured the percentage of nuclei that were MyHC^−^ or MyHC^+^ in mononucleated myotubes (<2), the % of nuclei that were inside myotubes containing between 2 and 5 nuclei (2 << 5) and the % of nuclei inside myotubes containing more than 5 nuclei (>5). Data are represented as average ± SEM, (*n* = 3). Statistical significance tested by one-way ANOVA; *p < 0.05, **p < 0.01.

We first monitored the intrinsic differentiation potential of these cell types in culture. MuSC ability to differentiate into MyHC-positive myotubes was comparable irrespective of whether they were isolated from wild type, young or old mdx mice (Fig 1A). Likewise, the adipogenic potential of FAPs was detected in all three conditions, with a more pronounced ability to form Oil red O-positive fat cells observed in young mdx mice (Fig 1B). These results indicate that changes in the intrinsic differentiation potential of MuSCs and FAPs unlikely accounts for the decline of the regeneration potential typically observed at advanced stages of disease progression in mdx mice. They rather suggest that an altered environment is responsible for the functional exhaustion of MuSCs in old mdx mice. Joe et al, have reported on the ability of FAPs to promote myogenesis indirectly, through functional interactions with MuSCs (Joe et al, 2010). Transwell co-culture experiments demonstrate that FAPs isolated from young mdx mice could potentiate the ability of MuSCs isolated from the same animals to form MyHC-positive multinucleated myotubes (Fig 1C); by contrast, FAPs isolated from old mdx mice repressed the formation of multinucleated myotubes from MuSCs isolated from young mdx mice (Fig 1C). Collectively, these results indicate that the intrinsic myogenic potential of MuSCs is not altered during disease progression in mdx mice, and that the progressive failure of MuSCs to support regeneration in dystrophic muscles of old mdx mice at late stages of disease is determined by extrinsic changes in muscle environment that compromise productive interactions between FAPs and MuSCs.

### The beneficial effects of HDACi are restricted to young mdx mice and correlate with a highly regenerative environment and with the functional status of FAPs

**Figure 2:**
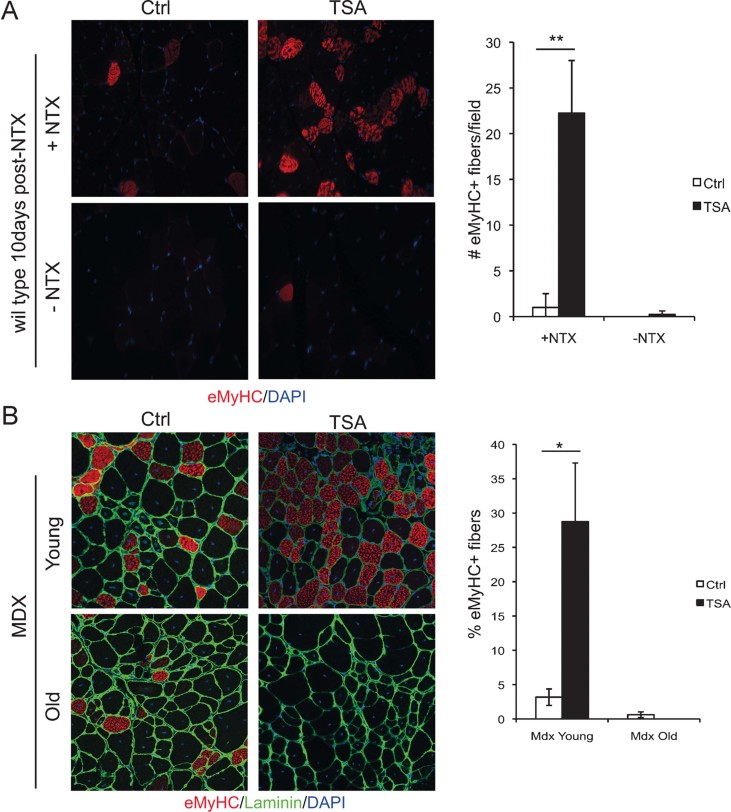
HDACi promote skeletal muscle regeneration only in actively regenerating muscles. A. Left panel: Right tibialis anterior of wild type mice were injured with notexin (+NTX) and the controlateral was left uninjured (−NTX). After 10 days of treatment with vehicle (Ctrl) or TSA, muscles were transverse sectioned and stained with an antibody against embryonal MyHC, (eMyHC – red) and nuclei counterstained with DAPI (blue). Right panel: Graph showing the % of eMyHC-positive myofibres. Data are represented as average ± SEM. *n* ≥ 3. Statistical significance assessed by *t*-test, **p < 0.01. B. Left panel: immunofluorescence staining for eMyHC (red) and laminin (green) of tibialis anterior transverse sections of young (2 months old) and old (1 year old) mdx mice treated for 15 days with vehicle (Ctrl) or TSA. Nuclei were counterstained with DAPI (blue). Right panel: graph showing the % of eMyHC-positive myofibres. Data are represented as average ± SEM. *n* ≥ 3. Statistical significance assessed by *t*-test, *p < 0.05.

Previous studies indicated that changes in muscle environment influence the ability of HDACi to promote regeneration, with a stage-dependent effect of HDACi on skeletal myogenesis *in vitro* and *in vivo* (Iezzi et al, 2002, 2004). However, the identity of the cell types that influence the effect of HDACi on muscle regeneration is unknown. To address this issue, we first evaluated the impact of muscle environment on HDACi ability to promote regeneration, by comparing the effect of HDACi on regenerating muscles *versus* unperturbed muscles. The HDACi Trichostatin A (TSA – 0.5 mg/kg) was administered to wild type mice in which right tibialis anterior muscle was induced to regenerate by injury, while non-injured contro-lateral left tibialis anterior muscle was used as control. After 10 days post-injury the regeneration process in injured muscles is largely resolved with few remaining regenerating embryonic MyHC (eMyHC)-positive fibres, which reflect the formation of regenerating muscle (Fig 2A). Exposure to TSA dramatically increased the number of eMyHC-positive myofibres only in injured muscles, while no significant effect was observed in non-injured, control muscles (Fig 2A). This finding indicates that TSA promotes and extends the regeneration process only in regenerating muscles. Likewise, TSA treatment increased the number of eMyHC-positive myofibres in muscles of young mdx mice, which are typically undergoing compensatory regeneration, but the same effect was not observed in old mdx mice, whose muscles have exhausted the regeneration potential (Fig 2B).

**Figure 3:**
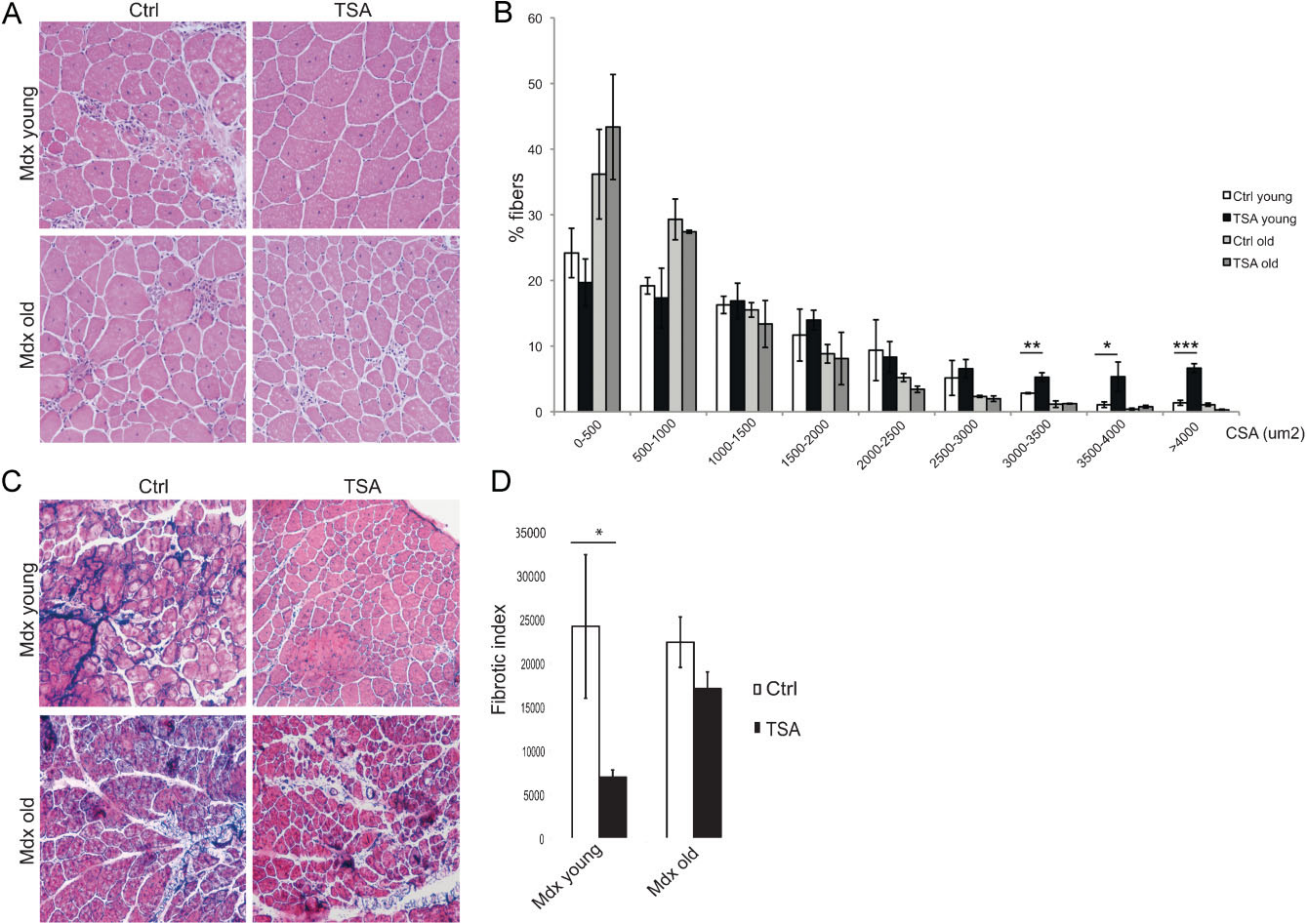
Skeletal muscles from aged MDX mice are resistant to HDACi-induced beneficial effects. A. Representative images of Hematoxylin and Eosin staining of tibialis anterior transverse sections of young (1.5 months old) and old (1 year old) mdx mice treated for 45 days with vehicle (Ctrl) or TSA (0.5 mg/kg). B. Graph of the analysis of myofibre cross sectional area (CSA) of muscles represented in (A) showing that TSA increases the caliber of myofibres only in young MDX muscles. Data are represented as average ± SEM. *n* ≥ 3. Statistical significance assessed by *t*-test, *p < 0.05, **p < 0.01, ***p < 0.01. C. Representative images of Masson's trichrome staining of tibialis anterior transverse sections of young (1.5 months old) and old (1 year old) MDX mice treated for 45 days with vehicle (Ctrl) or TSA (0.5 mg/kg). D. Graph representing the fibrotic index (quantification of collagen deposition) measured as blue area (reported as pixel^2^) per field. Data are presented as the average ± SEM. *n* ≥ 3. Statistical significance assessed by *t*-test, *p < 0.05.

The results described above indicate the importance of a permissive environment within regenerating muscles for the beneficial action of HDACi on dystrophic muscles. Indeed, previous studies have shown the therapeutic efficacy of HDACi when administered to young mdx mice; however, whether the same beneficial effects could be observed in mdx mice at later stages of disease progression has not been tested. To address this issue, we compared the effect of TSA treatment (0.5 mg/kg) for 45 days in young (1.5 months old) *versus* old (12 month old) mdx mice. TSA is a potent HDACi, with short half-life *in vivo*; however, we previously showed that continuous exposure to TSA by daily administration in mdx mice is sufficient to cause histone hyperacetylation at levels comparable to those observed with other HDACi, such as Butyrate, Valproic Acid (Minetti et al, 2006), and MS-275 (Colussi et al, 2008). TSA increased the cross sectional area (CSA) in young, but not in old mdx mice (Fig 3A and B). Likewise, TSA treatment could prevent the formation of fibrotic scars in young mice; however, it could not significantly affect the fibrosis developed in muscles of old mdx mice (Fig 3C and D). This evidence reveals a previously unappreciated stage-dependent effect of HDACi in mdx mice. Thus, HDACi ability to promote regeneration and inhibit fibrosis in muscles of mdx mice depends on a regeneration-permissive environment, which correlates with the competence of FAPs to support MuSC-mediated formation of myotubes (see Fig 1C).

### *Ex vivo* and *in vivo* evidence of stage-dependent repression of the adipogenic potential of FAPs and enhancement of functional interactions between FAPs and MuSC by HDCAi in mdx mice

**Figure 4:**
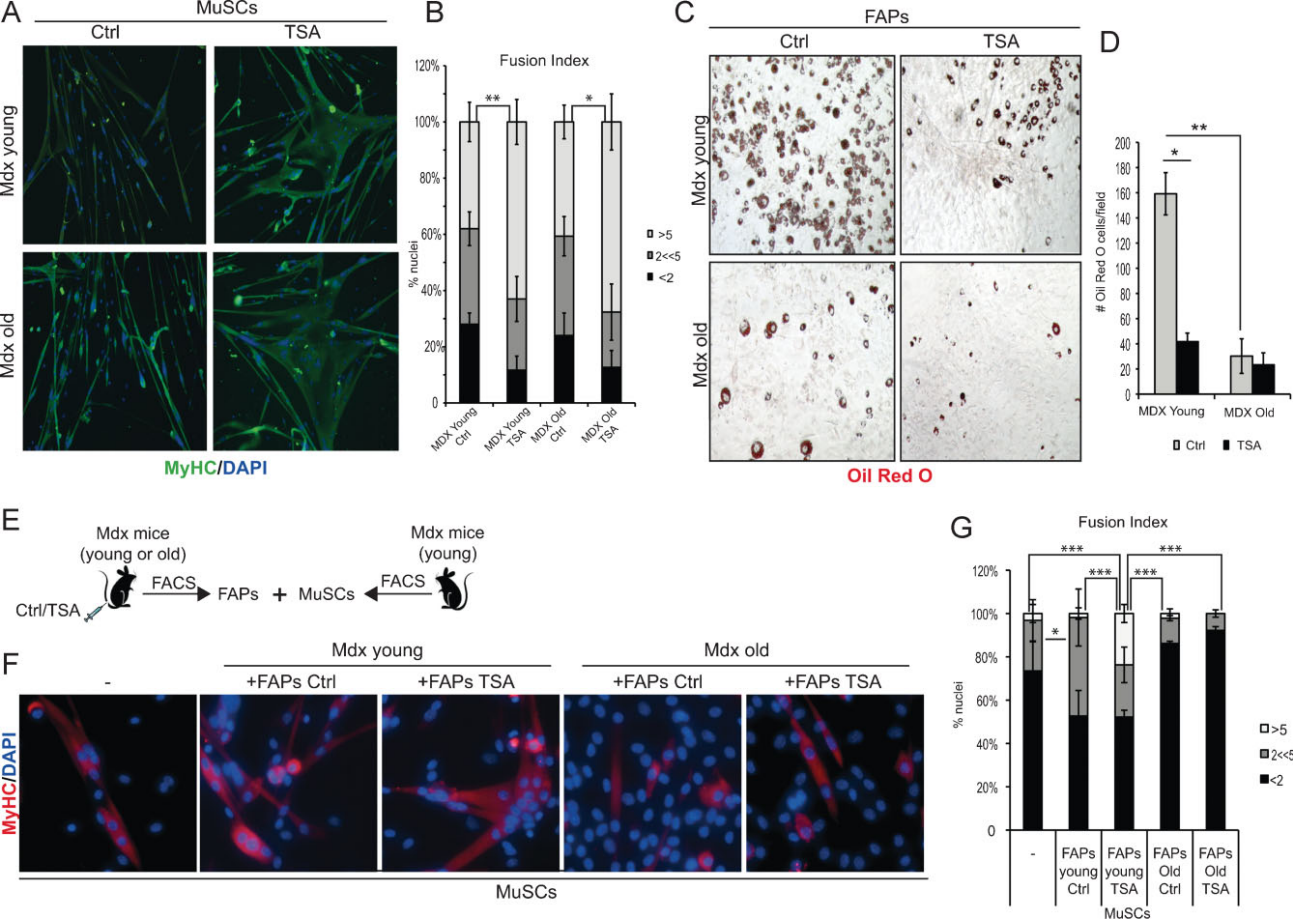
Intrinsic differentiation potential and functional interactions between MuSCs and FAPs in response to HDACi in MDX mice at different stages of disease. A. MuSCs isolated from young – 1.5 month old (upper panels) – and old – 1 year old (bottom panels) – MDX mice treated with ctrl or TSA (0.5 mg/kg) for 15 days, were isolated by FACS and cultured *in vitro* for 7 days in growth medium (GM) and then induced to differentiate in differentiation medium (5% HS) for further 3 days. Myogenic differentiation was assessed by MyHC immunofluorescnece. Representative images of MyHC (green) staining are shown. Nuclei were counterstained with DAPi (blue). B. Graph showing the fusion index of the experiment represented in (A). We measured the percentage of nuclei that were MyHC^−^ or MyHC^+^ in mononucleated myotubes (<2), the % of nuclei that were inside myotubes containing between 2 and 5 nuclei (2 << 5) and the % of nuclei inside myotubes containing more than 5 nuclei (>5). Data are represented as average ± SEM, *n* ≥ 2. Statistical significance tested by one-way ANOVA, *p < 0.05, **p < 0.01. C. FAPs isolated from young – 1.5 month old (upper panels) – and old – 1 year old (bottom panels) – MDX mice treated with ctrl or TSA (0.5 mg/kg) for 15 days, were isolated by FACS and cultured *in vitro* for 7 days in growth medium (GM) and then induced to differentiate in adipogenic differentiation medium (adp-DM) for further 6 days. Adipogenic differentiation was assessed by Oil red staining. Representative images are shown. D. Graph of the quantification of the number of Oil red O^+^ adipocytes per field in the same conditions showed in (C). Data are represented as average ± SEM of two different experiments (*n* ≥ 2); statistical significance assessed by *t*-test, *p < 0.05, **p < 0.01. E. Scheme: MuSCs FACS-isolated from young MDX mice were co-cultured in transwell with FAPs isolated from either young MDX mice (ctrl or TSA treated for 15 days) or old MDX mice (ctrl or TSA treated for 15 days). F. After 7 days of transwell co-culture the myogenic differentiation of satellite cells was assessed by immunostaining for MyHC (red). Nuclei were counterstained with DAPI (blue). G. Graph showing the fusion index of the experiment represented in (F). We measured the percentage of nuclei that were MyHC^−^ or MyHC^+^ in mononucleated myotubes (<2), the % of nuclei that were inside myotubes containing between 2 and 5 nuclei (2 << 5) and the % of nuclei inside myotubes containing more than 5 nuclei (>5). Data are represented as average ± SEM, *n* ≥ 2. Statistical significance tested by one-way ANOVA, *p < 0.05, **p < 0.01, ***p < 0.001.

The results described so far emphasize the importance of reciprocal interactions between different cell types to generate a microenvironment conducive for HDACi-mediated regeneration of dystrophic muscles. Previous works demonstrated that MuSC interactions with FAPs regulate the ability of skeletal muscle to undergo either regeneration or fibroadipogenic degeneration (Joe et al, 2010; Rodeheffer, 2010; Uezumi et al, 2010) and suggest that FAPs/MuSCs interactions might also influence the stage-specific response to HDACi in mdx mice (see Figs 1 and 2). To further determine the importance of functional interactions between FAPs and MuSCs for the beneficial effects of HDACi on mdx mice, we analysed the intrinsic differentiation potential and the functional interactions between these cells types in response to HDACi under different environmental contexts. MuSCs and FAPs were isolated from mdx mice young or old, treated or not with TSA. Exposure to TSA did not substantially alter the FACS profiles in all the experimental conditions, as compared to untreated animals (Supporting Information Fig S2). Moreover, MuSCs isolated from young or old mdx mice treated with TSA displayed the same ability to form large multinucleated myotubes in culture, upon incubation in differentiation medium (DM; Fig 4A and B). This indicates that the intrinsic ability of MuSCs to respond to HDACi is equivalent in young and old mdx mice, once insulated from their *in vivo* regenerative context, despite the fact that old mdx mice are resistant to the beneficial effect of HDACi (Figs 2 and 3). By contrast, FAPs isolated from young mdx mice exposed to TSA showed a dramatic reduction in the ability to differentiate into Oil red-positive adipocytes, whereas the adipogenic potential of FAPs isolated from old mdx mice exposed to TSA was unaltered (Fig 4C and D). The different response to HDACi of FAPs derived from young and old mdx mice, correlates with the regeneration permissive (young mdx mice) *versus* repressive (old mdx mice) muscle environment, and suggests that FAP commitment to the fibro-adipogenic lineage is pharmacologically reversible in regenerating muscles of young mdx mice, but becomes constitutive in old mdx mice. To further demonstrate the importance of regeneration cues in HDACi-mediated inhibition of FAP differentiation, we analysed the adipogenic potential of FAPs isolated from regenerating muscles *versus* unperturbed muscles (from the same experiment shown in Fig 2). Supporting Information Fig 3 shows that exposure of mice to TSA reduced the adipogenic potential of FAPs from regeneration (notexin-injured), but not from unperturbed muscles. This evidence further supports the notion that regeneration cues are required for HDACi-mediated inhibition of FAP adipogenic potential, and this correlates with the ability of HDACi to promote muscle regeneration (as shown in Fig 2).

Collectively, the results shown above indicate that the different ability of TSA to promote beneficial effects in young, but not in old mdx mice coincides with their ability to repress the fibroadipogenic potential of FAPs derived from a regenerative environment. This data suggests that FAPs could be the cellular determinants of TSA effects on mdx mice, and provide the rationale to test whether changes in FAPs from old mdx mice negatively affect the regeneration potential of MuSCs and their ability to respond to TSA *in vivo*. To this purpose, we used the transwell co-culture assay to assess the effect of FAPs isolated from young or old mdx mice that were exposed or not to TSA, on MuSC ability to differentiate into myotubes (see scheme in Fig 4E). FAPs isolated from muscles of young mdx mice potentiated the differentiation ability of MuSCs, and FAPs isolated from muscles of TSA-treated young mdx mice further implemented this activity, leading to the formation of larger, multinucleated myotubes from co-cultured MuSCs (Fig 4E and F). Consistently, the transcripts of myogenic genes, such as MhC3, MhC8 and MCK, were increased in MuSCs co-cultured with FAPs isolated from muscles of TSA-treated young mdx mice (Supporting Information Fig S4). By contrast, FAPs isolated from muscles of old mdx mice, treated or not with TSA, impaired the differentiation ability of MuSCs (Fig 4F and G). Thus, while the intrinsic differentiation potential of MuSCs appears unaltered during mdx disease progression, co-culture experiments reveal the key role of FAPs in transmitting to MuSCs either signals from an environment permissive for response to TSA or inhibitory signals that confer resistance to TSA in aged mdx muscles. These results indicate that indirect interactions with FAPs derived from different environments modulate the regenerative ability of MuSCs and their response to HDACi *in vivo*.

**Figure 5:**
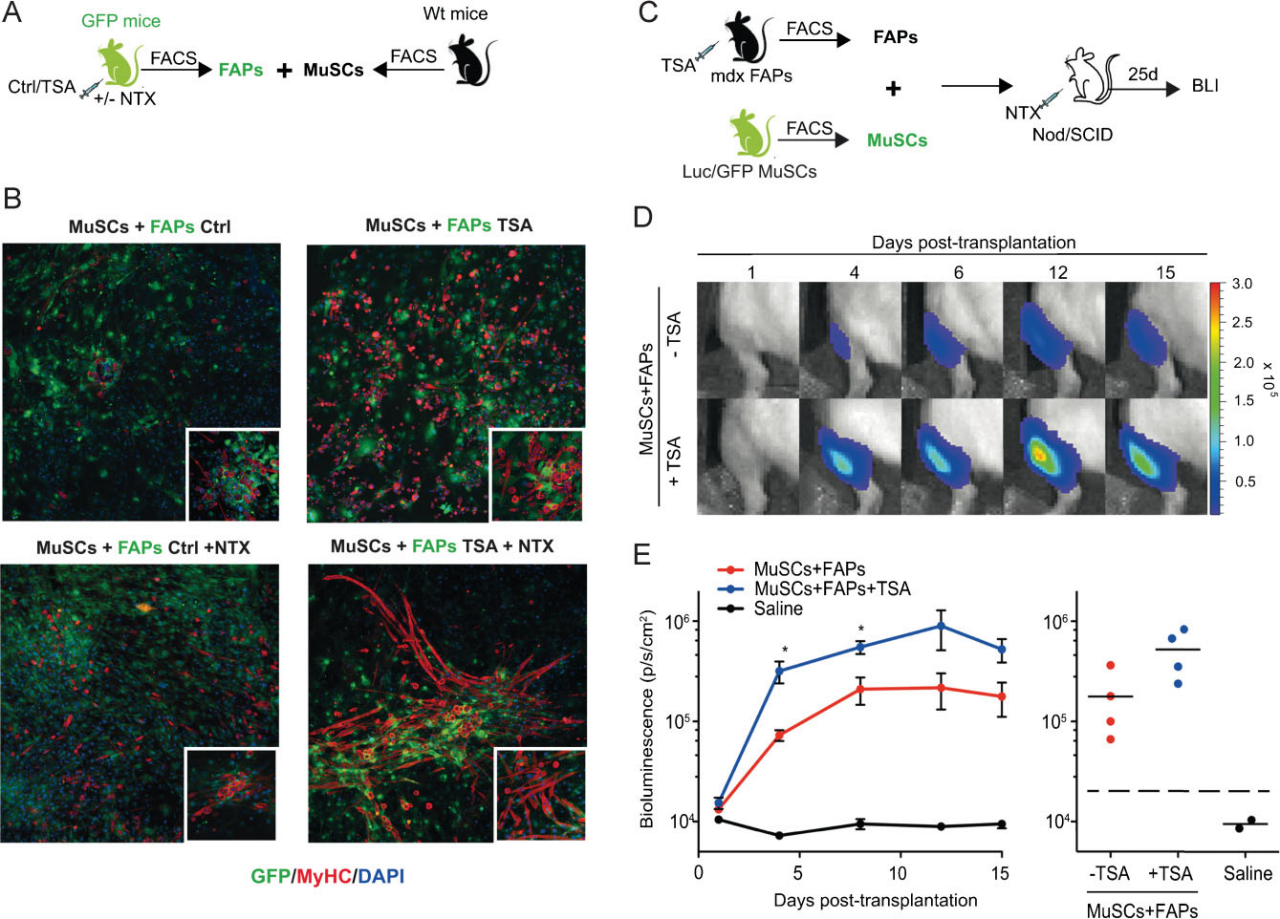
HDACi enhance the ability of FAPs to promote myogenic activity of MuSCs on injured muscles *ex vivo* and *in vivo.* A. Schematic representation of co-culture strategy. GFP – FAPs were isolated from notexin injured (+NTX) or non-injured (ctrl) skeletal muscles of GFP mice (*n* = 2), treated with vehicle (ctrl) or TSA for 5 days, and then co-cultivated for 14 days with MuSCs isolated from wt mice. B. Representative images of immunostaining for MyHC (red) and GFP (green) of co-culture between FAPs from ctrl (left panel) and TSA (right panel) GFP mice and wt MuSCs. Insets show a higher magnification. C. Schematic representation of MuSCs/FAPs co-transplantation into the irradiated tibialis anterior of immunodeficient Nod/SCID mice. MuSCs were isolated from double-transgenic Luciferase-EGFP mice. FAPs were isolated from mdx mice treated with TSA or control (vehicle) for 5 days. Twenty-four hours prior to intramuscular MuSCs/FAPs co-transplantation, recipient mice received local tissue injury via intramuscular NTX injection in the tibialis anterior. D. Non-invasive bioluminescence imaging was used to monitor the effect of FAPs on MuSC proliferation *in vivo*. 1000 Luc/GFP MuSC were co-transplanted with 1000 FAPs from mdx mice previously treated with TSA or control. Images were taken up to 15 days post-transplantation and luminescence quantified. E. Plots of bioluminescence data from (D) represented in photons/s/cm^2^ as an average ± SEM and individually (*n* = 4, *p < 0.05).

To further evaluate the effect of the regenerative environment on functional interactions between FAPs and MuSCs, we used *ex vivo* co-culture assays with FAPs isolated from muscles of GFP-mice, either unperturbed or injured, and treated or not with TSA, and MuSCs from wild type mice (Fig 5A). TSA slightly promoted the functional interactions between FAPs from non-injured muscles and adjacent MuSCs (Fig 5B – compare upper panels), and dramatically enhanced the ability of FAPs from injured muscles to promote myotube formation from co-cultured MuSCs (Fig 5B – compare bottom panels). We next investigated the effect of the regenerative environment on functional interactions between FAPs and MuSCs *in vivo* by co-transplantation experiments. FAPs were isolated from regenerating muscles of young mdx mice, either treated or not with TSA, and transplanted into muscles of NOD/SCID mice together with MuSCs isolated from muscles of luciferase/GFP mice (Fig 5C). The proliferative behaviour of Luc/GFP MuSCs was monitored over time by non-invasive bioluminescence imaging, as previously described (Sacco et al, 2008). The luciferase signal revealed successful colonization and engraftment of MuSCs into the muscles of recipient mice. This setting was instrumental to determine whether TSA exposure could “prime” FAPs from regenerating muscles to support the ability of co-transplanted MuSCs to regenerate injured muscles in recipient mice. The luciferase signal was monitored in living mice at different time points after injection, as readout of the effect of TSA on functional interactions between co-transplanted cells. Figure 5D shows that FAPs from TSA-treated mdx mice enhanced the luciferase signals from co-transplanted MuSCs during the first 2 weeks of treatment, and the effect persisted in the following days (Fig 5D and E).

### FAPs from regenerating muscles restore HDACi ability to promote regeneration in old mdx mice

**Figure 6:**
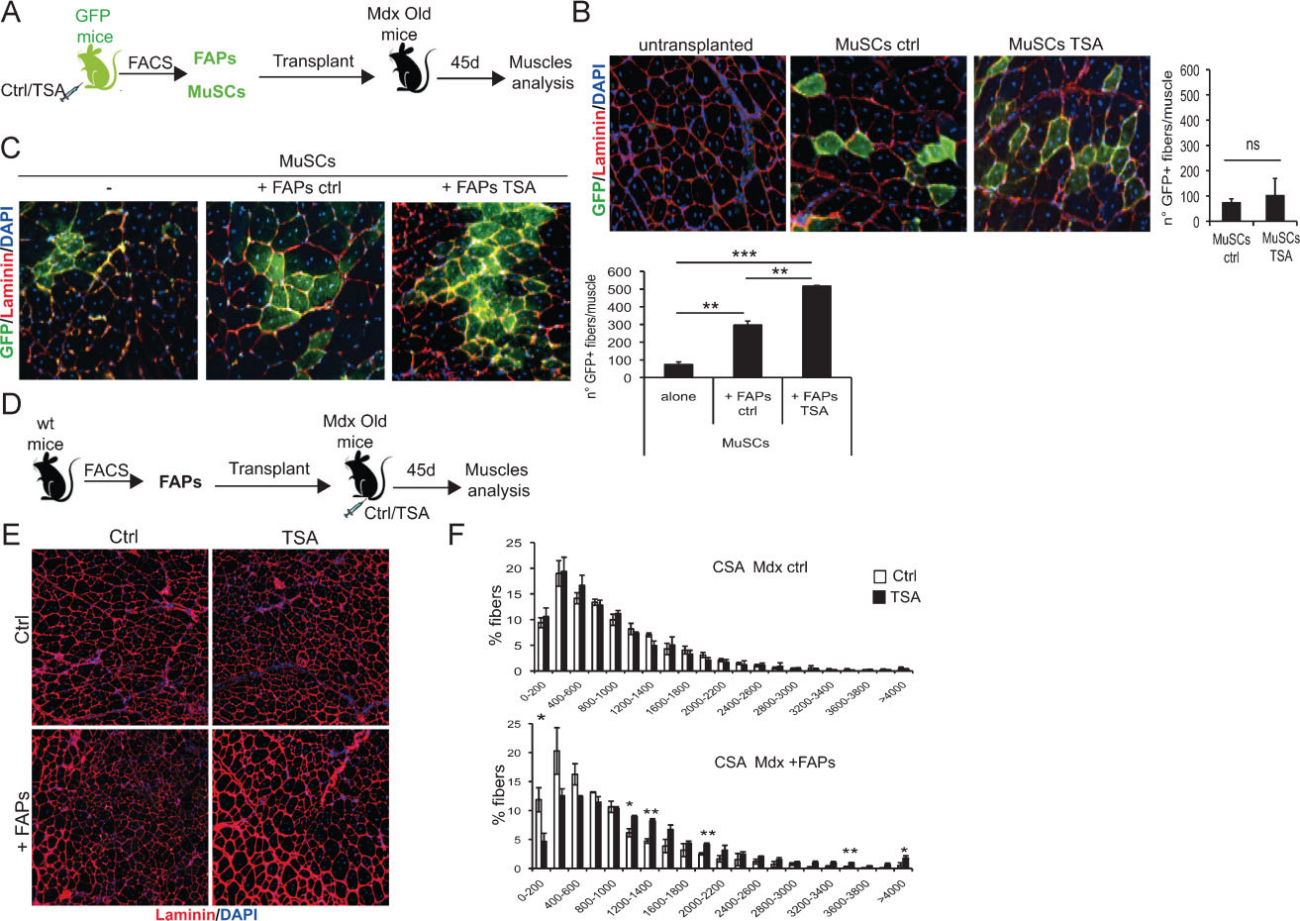
FAPs from HDACi-treated mice enhance the *in vivo* differentiation potential of MuSCs and restore the responsiveness of aged MDX muscles to HDACi beneficial effects. A. Schematic representation of transplant strategy. Skeletal muscles of GFP mice were injured by notexin (NTX) injection. Injured GFP mice were treated with TSA or vehicle (ctr). After 5 days of treatment FAPs and MuSCs were isolated by FACS and 30,000 GFPpos cells of each population were transplanted into the tibialis anterior (pre-injured with NTX 1 day before) of recipient 1-year-old MDX mice, either alone or in co-tranplantation (FAPs + MuSCs). Three weeks after, transplanted muscles were isolated and processed for staining with antibodies against GFP (green) and laminin (red). Nuclei were counterstained with DAPI (blue). B. Left panels: representative images of engrafted areas in muscles transplanted with ctrl and TSA MuSCs. Right panel: quantification of the total number of GFP^+^ myofibres per muscle. Data are represented as average ± SEM. *n* = 3. C. Representative images of engrafted areas in muscles transplanted with ctrl MuSCs alone (left panel) or co-transplanted with FAPs ctrl (mid panel) and FAPs TSA (right panel). Quantification of the total number of GFP^+^ myofibres per muscle shows that co-transplantation with FAPs-ctrl significantly increases the number of GFP^+^ myofibres. This number is further increased when MuSCs are co-transplanted with FAPs isolated from TSA treated mice. Data are represented as average ± SEM. *n* = 3. Statistical significance tested by one-way ANOVA, **p < 0.01. ***p < 0.001. D. Schematic representation of transplant strategy. 30,000 FACS-isolated FAPs from skeletal muscles of 2-month old wt mice were transplanted into the right tibialis anterior (pre-injured with NTX 1 day before) of recipient 1-year-old MDX mice, while the controlateral was left untransplanted. Recipient mice were then treated with vehicle (Ctrl) or TSA for 45 days. Transplanted and non-transplanted muscles were harvested and immunostained with antibody against laminin (red) and nuclei counterstained with DAPI (blue). E. Representative images of muscles untransplanted (ctrl, upper panels) and transplanted with FAPs (+FAPs, bottom panels), either ctrl (left panels) and TSA (right panels) treated as described in (D). F. Graph of the analysis of myofibre cross sectional area (CSA) of muscles represented in (E). Upper graph represents the CSA of untransplanted muscles (Mdx Ctrl); bottom graph shows CSA of transplanted muscles (+FAPs). Data are represented as average ± SEM. *n* = 2. Statistical significance assessed by *t*-test, *p < 0.05. **p < 0.01.

To directly link the stage-specific effect of HDACi on mdx mice to the activity of FAPs, we tested whether co-transplantation of FAPs from TSA-treated wild type injured mice could enhance MuSC ability to colonize old mdx muscles (see schematic representation in Fig 6A); and if FAPs from young regenerating muscles could restore the TSA ability to promote an increase of muscle size in old mice (see schematic representation in Fig 6D).

Transplantation of MuSCs isolated from injured muscles of GFP mice previously exposed to either TSA or control vehicle led to engraftment in muscles of old mdx mice with comparable efficiency (Fig 6B). Co-transplantation of FAPs isolated from injured muscles of wild type mice increased the engraftment efficiency of MuSCs from GFP mice into old mdx muscles (Fig 6C). Importantly, co-transplantation of FAPs isolated from injured muscle of TSA-treated mice further enhanced GFP-MuSCs engraftment into muscles of old mdx mice (Fig 6C). This data indicates that muscles of old mdx mice provide an environment unfavourable to the engraftment of transplanted MuSCs, and FAPs isolated from regenerating muscles exposed to TSA can implement the engraftment ability of MuSCs in old mdx mice. Collectively, data shown in Fig 6A–C demonstrate that FAPs exposed to TSA and regeneration cues promote muscle regeneration via functional interactions with MuSCs. Thus, we tested whether transplantation of FAPs isolated from injured muscles of young mice could restore the ability of TSA to promote endogenous regeneration of muscles in old mdx mice (see schematic representation of the experiment in Fig 6D). Figure 3 showed that old mdx mice display a remarkable resistance to the pro-regenerative activity of TSA (as measured by increased CSA), which is typically observed in young mdx mice (see Fig 3; Minetti et al, 2006). Transplantation of FAPs isolated from young regenerating muscles could not promote an increase in CSA of old mdx mice treated with control vehicle; however, these FAPs restored the response of old mdx muscles to TSA (Fig 6E and F). Indeed, old mdx muscles transplanted with FAPs isolated from young muscles responded to 45-day exposure to TSA with an increase in CSA (Fig 6F) that was comparable to that observed in TSA treated young mdx mice (see Fig 3).

### Follistatin is soluble mediator of functional interactions between FAPs and MuSCs from mdx mice exposed to HDACi

**Figure 7:**
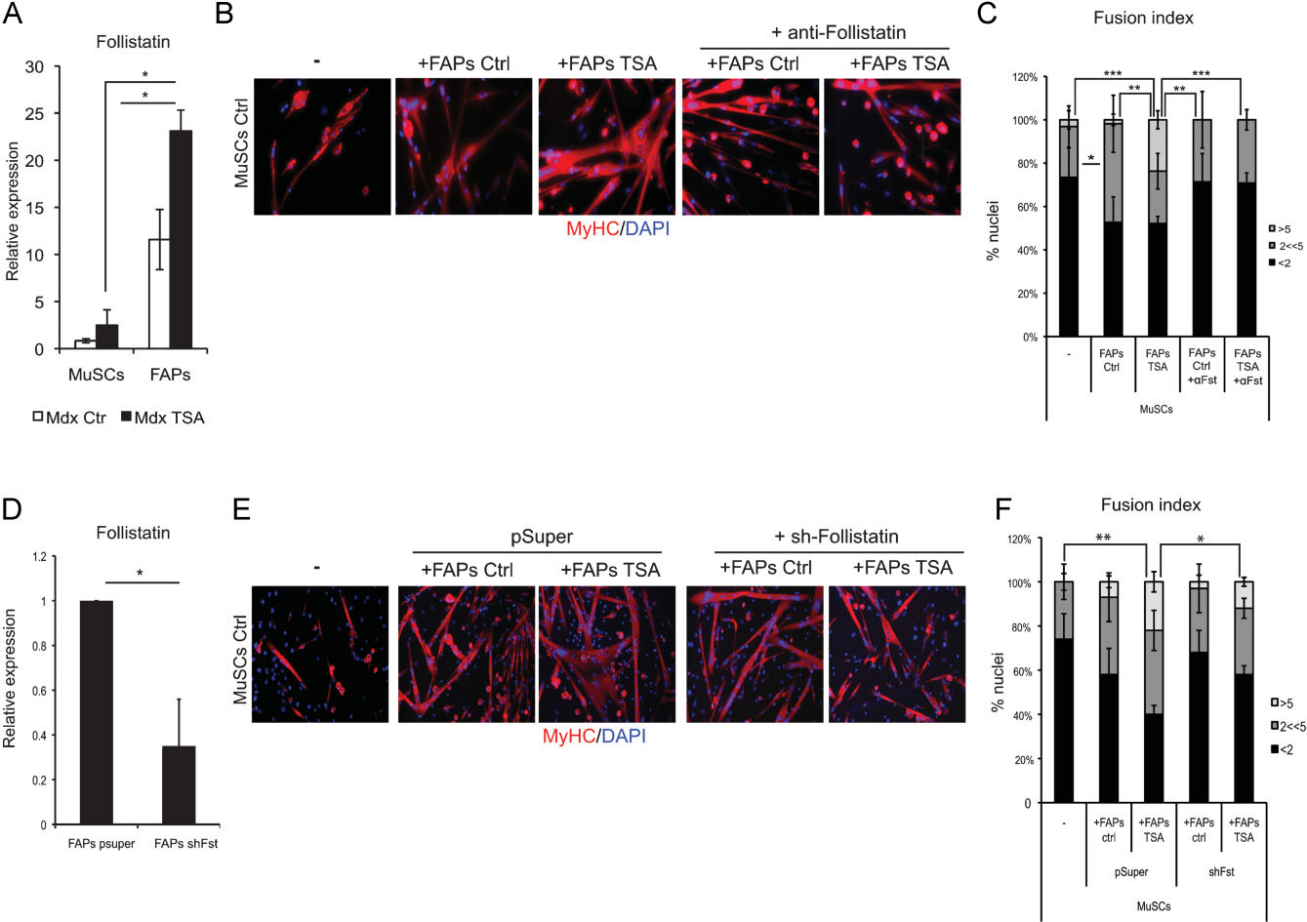
Follistatin is soluble mediator of functional interactions between FAPs and MuSCs from mdx mice exposed to HDACi. A. FAPs and MuSCs were isolated from 1.5-month old mdx mice ctrl- and TSA-treated for 15 days. Cells were cultured for 7 days in growth medium (GM) and then Follistatin RNA levels were analysed by qRT-PCR. Follistatin is preferentially expressed by FAPs, as compared to MuSCs, and is strikingly up-regulated in FAPs isolated from TSA-treated mdx muscles. B. MuSCs from mdx mice and FAPs, from either mdx ctrl or TSA treated (15 days) mice, were co-cultured in transwell chambers (pore of 1 µm). FAPs were plated on the transwell insert and MuSCs were plated on the bottom compartment. After 7 days of co-cultures in the presence or absence of neutralizing antibodies against Follistatin, MuSCs were immunostained for MyHC (red) and nuclei counterstained by DAPI (blue). C. Graph showing the fusion index of the experiment represented in (B). We measured the percentage of nuclei that were MyHC^−^ or MyHC^+^ in mononucleated myotubes (<2), the % of nuclei that were inside myotubes containing between 2 and 5 nuclei (2 << 5) and the % of nuclei inside myotubes containing more than 5 nuclei (>5). Data are represented as average ± SEM, *n* ≥ 2. Statistical significance tested by one-way ANOVA, **p < 0.01, ***p < 0.001. D. Follistatin RNAi in FAPs from ctrl mdx mice was performed by transfection of a shRNAi against follistatin (shFollistatin) and pSuper was used as ctrl. Graph shows the RNA levels of follistatin measured by qRT-PCR in FAPs upon RNAi. E. FAPs were treated (24 h) with TSA or not (ctrl) *in vitro* and subsequently co-cultured for 5 days in transwell, as described in B, with MuSCs isolated from mdx mice. MuSCs were then immunostained for MyHC (red) and nuclei counterstained by DAPI (blue). F. Graph showing the fusion index of the experiment represented in (E). We measured the percentage of nuclei that were MyHC^−^ or MyHC^+^ in mononucleated myotubes (<2), the % of nuclei that were inside myotubes containing between 2 and 5 nuclei (2<<5) and the % of nuclei inside myotubes containing more than 5 nuclei (>5). Data are represented as average ± SEM, *n* ≥ 2. Statistical significance tested by one-way ANOVA, *p < 0.05, **p < 0.01.

In search for a soluble mediator of HDAC-induced functional interactions between FAPs and MuSCs, we considered follistatin as a potential candidate, as our previous studies showed that follistatin is up-regulated by treatment with HDACi (Iezzi et al, 2004; Minetti et al, 2006). When we compared the basal levels of follistatin transcripts in MuSCs and FAPs isolated from young mdx mice, we observed that FAPs expressed 10-fold higher levels of follistatin than MuSCs, with a twofold increase induced by TSA treatment (Fig 7A). In transwell co-culture experiments, incubation of FAPs with neutralizing antibodies against follistatin significantly impaired their ability to promote the formation of multinucleated myotubes from MuSCs, both in normal conditions or after exposure to TSA (Fig 7B and C). Knock-down of follistatin in FAPs by shRNAi (Fig 7D) reduced the ability of TSA to stimulate the formation of multinucleated myotubes from MuSCs in transwell co-culture experiments, with a similar efficiency as that observed with neutralizing antibodies (compare Fig 7E and F with Fig 7B and C), supporting the conclusion that FAP-derived follistatin is a mediator of functional interactions between FAPs and MuSCs.

## DISCUSSION

The transition from compensatory regeneration to fibroadipogenic degeneration is a crucial event in the pathogenesis of MDs. Despite the functional exhaustion of MuSCs has been implicated in the progressive decline of the regeneration potential of dystrophic mice (Blau et al, 1983; Sacco et al, 2010), the cellular and molecular effectors of the regeneration/degeneration switch remain unknown. This gap of information precludes effective strategies toward implementing the regeneration activity at the expense of fibro-adipogenesis.

Here we show that changes in FAP activity during disease progression contribute to the exhaustion of muscle regeneration and to the progressive fibroadipogenic degeneration of dystrophic muscles, and determine the responsiveness of mdx mice to HDACi. Our data revealed an unexpected stage-specific effect of HDACi in mdx mice, and indicate that FAPs transmit to satellite cells changes in the muscle environment during the disease progression. In the permissive regenerative environment of young mdx mice FAPs support satellite cell differentiation and are highly sensitive to HDACi-mediated inhibition of their adipogenic potential. This correlates with the ability of HDACi to promote muscle regeneration and inhibit fibro-adipogenic degeneration in young mdx mice. At late stage of disease progression (old mdx mice) FAPs constitutively inhibited satellite cell differentiation and become resistant to HDACi. This evidence indicates that FAP phenotype and function are highly influenced by the environmental changes occurring in dystrophic muscle during disease progression. Importantly, FAPs from regenerating muscles, such as those of young mdx mice, restored the ability of HDACi to promote satellite cell-mediated formation of myotubes and endogenous muscle regeneration in old mdx mice. Thus, FAPs appear to be central cellular determinants of disease progression in mdx mice and influence the response of dystrophic muscles to pharmacological interventions that promote endogenous regeneration and inhibit fibro-adipogenic degeneration. However, it should be stressed that fibrosis is a complex event, presumably resulting from an interplay between distinct pathways and signals (Serrano et al, 2011) and future studies should clarify the relative contribution and cooperation of FAPs and other fibrogenic pathways.

As FAPs were isolated in this study as Sca1^pos^ cells they likely represent an extremely heterogeneous population of cells that might include a variety of cell types previously implicated in muscle regeneration, such as PW1 interstitial cells (PICs; Mitchell et al, 2010) and vessel-derived pericytes (Dellavalle et al, 2011). Thus, it is formally possible that these, and possibly other cell types, might contribute to the observed ability of FAPs to promote MuSC activity in co-culture. However, it is unlikely that PICs and pericytes are the cellular target of TSA ability to reduce fibro-adipogenic degeneration in mdx mice, as these cells have not been shown to posses any fibro-adipogenic potential in previous studies (Dellavalle et al, 2011; Mitchell et al, 2010). Further studies will be necessary to assign specific function to discrete sub-populations of Sca1^pos^ cells in muscles of mdx mice exposed to HDACi.

Overall, our data indicate that FAPs provide a novel source of endogenous, pharmacologically inducible, population of intramuscular cells that can be exploited to regenerate dystrophic muscles and prevent deleterious events, such as fibro-adipose infiltration of muscles that complicate DMD progression. The stage-specific functional behaviour of FAPs and response to HDACi encourage to extend future studies to the identification and characterization of human FAPs from muscles of DMD patients, as predictive biomarker of response to pharmacological interventions, such as treatment with HDACi, and possible target for strategies that could restore the regeneration potential in muscle of DMD patients at advanced stages of disease progression. As FAPs are isolated as Sca1^pos^ cells in mdx mice, but the human counterpart of Sca1 antigen is currently unknown, future efforts should be directed toward the identification of reliable markers that can allow the isolation of FAPs from human biopsies.

## MATERIALS AND METHODS

### Animals and *in vivo* treatments

Normal wild-type C57/BL6, C57Bl6 mdx mice and Nod/SCID mice were purchased from the Jackson Laboratories. Ubiquitin-GFP mice were a kind gift from G. Cossu; EGFP transgenic mice (a kind gift from Dr. Bill Stallcup) and a L2G85 (FLuc) strain ubiquitously expressing luciferase from the ACTB promoter were used to generate the double-transgenic animals. Animals were used at the specified age and treated for the indicated periods with daily i.p. injections of Trichostatin A, TSA (0.6 mg/kg/day; Sigma), dissolved in saline solution, or saline alone as control (Ctrl). Mice were bred and maintained according to the standard animal facility procedures, and all experimental protocols were approved by the internal Animal Research Ethical Committee according to the Italian Ministry of Health and complied with the NIH Guide for the Care and Use of Laboratory Animals.

### Cell preparation and FACS isolation

For fluorescence-activated cell sorting, hind limb muscles were minced and digested in HBSS (Gibco) containing 2 µg/ml Collagenase A (Roche), 2.4 U/ml Dispase I (Roche), 10 ng/ml DNase I (Roche), 0.4 mM CaCl_2_ and 5 mM MgCl_2_ for 90 min at 37°C. Cells were stained with primary antibodies (10 ng/ml) CD31-PacificBlue (Invitrogen), CD45-eFluor450 (eBioscience), Ter119-eFluor450 (eBioscience), CD11b-Pacific blue, Sca-1-FITC (BD Pharmingen), CD34-Biotin (eBioscience) and α7integrin-APC (kindly provided by Dr. Fabio Rossi) for 30 min on ice. A subsequent incubation, 30 min on ice, with Streptavidin-PE-Cy7 (1/500; BD Pharmingen) was performed. Cells were finally washed and resuspended in HBSS containing 0.2% w/v BSA and 1% v/v Penicillin–Streptomycin. Flow cytometry analysis and cell sorting were performed on a DAKO-Cytomation MoFlo High Speed Sorter.

Muscle satellite cells (MuSCs) were isolated as Ter119^−^/CD45^−^/CD31^−^/CD34^+^/α7-integrin^+^/Sca-1^−^ cells; FAPs cells were isolated as Ter119^−^/CD45^−^/CD31^−^/CD34^+^/α7-integrin^−^/Sca-1^+^ cells. FACS procedure in which CD11b was used in place of Ter119 gave rise to an identical population, with indistinguishable phenotype, biological activity and response to TSA.

### Culture conditions of FAPs and MuSCs

Freshly sorted cells were plated on 0.1% gelatin-coated dishes in BIOAMF-2 complete medium (ATGC) as a growth medium (GM). For adipogenic differentiation, after 7 days of GM, cells were exposed for 3 days to adipogenic induction medium consisting of DMEM with 10% FBS (Gibco), 0.5 mM IBMX (Sigma), 0.25 µM dexamethasone (Sigma) and 10 µg/ml insulin (Sigma), followed by further 3 days in adipogenic maintenance medium, consisting of DMEM with 10% FBS and 10 µg/ml insulin. For TSA *in vitro* treatment, cells were treated for 24 h with 50 nM TSA in GM and then switched in adipogenic DM without the drug. Cell culture inserts with 1.0-µm pore and 6-well culture plates (BD bioscience) were used for transwell co-culture. Inserts and plates were coated with 0.1% gelatin. 1 × 10^4^ freshly sorted MuSCs were plated in the bottom of the plate, while 1 × 10^4^ FAPs cells were plated on the upper insert. Transwell co-cultures were maintained in GM for 7 days and then harvested for analyses. Where specified, FAPs were exposed to a neutralizing antibody against Follistatin (R&D) at the final concentration of 4 µg/ml (Iezzi et al, 2004).

### RNA interference

Downregulation of Follistatin expression in FAPs was performed by transfection with lipofecatamin 2000 (Invitrogen) of a shRNAi against follistatin (shFollistatin; Iezzi et al, 2004), pSuper empty vector was used as ctrl.

### Muscle injury and transplantation experiments

Muscle injury was performed by intramuscular injection of 10 µg/ml notexin (NTX, Sigma). For transplantation experiments in Nod/SCID mice, recipient animals were anaesthetized via isoflurane inhalation and received 10 µl intramuscular injections into the tibialis anterior of either 10 µg/ml notexin for local tissue injury, or freshly isolated muscle stem cells (MuSC) and FAPs cells resuspended in PBS.

For transplantation experiments in mdx mice, recipient animals were anaesthetized via isoflurane inhalation and received intramuscular injections into the tibialis anterior of 10 µL NTX (10-5M) for local tissue injury. Twenty-four hours post injury freshly FACS isolated cells (MuSCs and/or FAPs) resuspended in PBS were then injected intramuscularly.

**The paper explained**

**PROBLEM**

This manuscript elucidates the role of distinct cellular components of the skeletal muscle repair machinery in the pathogenesis of Duchenne Muscular Dystrophy (DMD), and indicates the biological rationale for the pharmacological correction by HDAC inhibitors (HDACi) of a key pathogenic event in the disease progression – *i.e.*, the functional exhaustion of the regeneration potential of muscle satellite cells and the parallel fibroadipogenic degeneration.

**RESULTS**

The data demonstrate that a population of muscle interstitial cells that is distinct from satellite cells and has previously been defined to as FibroAdipogenic Progenitors (FAPs), contribute to DMD pathogenesis by progressively failing to support satellite cell-mediated regeneration of dystrophic muscles in mdx mice.

The functional interactions between FAPs and satellite cells are mediated by soluble factors, such as follistatin, a previously identified as a target of HDACi. The functional status of FAPs appears to determine the ability of HDACi to promote regeneration and prevent fibro-adipogenic degeneration in young, but not old, dystrophic muscles.

**IMPACT**

The information derived by this study will have an impact on the immediate translation of HDACi in the pharmacological treatment of DMD patients, as it indicates cellular targets and mechanism of action that will help to the identification of new biomarkers for patient selection in clinical trials and for monitoring the efficacy of the intervention.

### Histology and immunofluorescence

Tibialis anterior muscles were snap frozen in liquid nitrogen-cooled isopentane and then cutted transversally. Cryosections (8 µm) and cultured cells were fixed in 4% PFA for 20 min and permeabilized with 100% methanol for 6 min at −20°C. To avoid unspecific binding, muscle sections were first blocked with a solution containing 4% BSA in PBS and then with anti-mouse AffiniPure Fab fragment (Jackson, 1:100). Immunostaining with primary antibodies was performed overnight at 4°C. Antibody binding was revealed using species-specific secondary antibodies coupled to Alexa Fluor 488 or 543 (Molecular Probes). Nuclei were visualized by counterstaining with DAPI. Primary antibodies used were against: Laminin (Sigma; 1:100), embryonic MyHC (Developmental Studies Hybridoma Bank, DSHB; 1:20), MF-20 (DSHB, 1:20), MyoD (Santa Cruz, 1:20), GFP (Invitrogen, 1:500). To stain lipids, 10% formalin fixed cells and tissues were rinsed with water and then with 60% isopropanol, stained with Oil red O in 60% isopropanol and rinsed with water. Images were acquired with a Leica confocal microscope and edited using the Photoshop software. Fields reported in the figures are representative of all examined fields. For Hematoxylin and Eosin staining, cryosections were fixed in 4% PFA, then washed in PBS-1X and then they were stained in haematoxylin for 4 min and eosin for 6 min. Then the cryosections were dried in ethanol and at the end they were fixed in xylene and mounted with EUKITT mounting (O. Kindler GmbH & CO). To stain fibrotic tissue Masson's trichrome analyses was used. Muscle cryosections were stained in Working Weigert's Iron Hematoxilin Solution for 5 min, washed in running tap water for 5 min and stained in Biebrich Scarlet-Acid Fucsin for 5 min. Rinsed in de-ionized water, placed in Working Phosphotungstic\Phosphomolybdic acid solution for 5 min, stained in Aniline Blue solution for 5 min and in acid acetic 1% for 2 min. The slides were mounted with EUKITT mounting. For immunofluorescence after cell transplantation, tibialis anterior muscles were excised and frozen prior to the collection of transverse muscle sections using a Leica CM 3050S cryostat. To identify donor-derived GFP^+^ myofibres, sections were incubated in the following primary antibodies: rabbit anti-GFP (Invitrogen), rat anti-laminin (Millipore) and Hoechst 33258 (Invitrogen) for the identification of nuclei.

Images were acquired using an inverted fluorescent microscope (Nikon TE300), 10× objective lens, CCD SPOT RT camera and SPOT imaging software (Diagnostic Instruments, Inc.). Images were composed, edited and modifications applied to the whole image using Photoshop CS4 (Adobe).

### Quantitative analysis

The cross-sectional area (CSA) was calculated using the ImageJ software downloaded from http://rsb.info.nih.gov/ij. Fibrotic areas were measured by selecting four representative and non-adjacent sections and photographing up to three microscopic fields. The total fibrosis was calculated from sections evaluating image analysis algorithms for colour deconvolution. ImageJ was used for image processing, the original image was segmented with three clusters and the plugin assumes images generated by colour subtraction (white representing background, blue collagen, and magenta non collagen regions; (Krajewska et al, 2009; Ruifrok & Johnston, 2001). Oil Red O areas were quantified by selecting four representative and non-adjacent sections and photographing up to three microscopic fields. Oil red O areas were measured by using ImageJ, calculating the area of red pixels (pixel^2^) per field. To assess the efficiency of transplantation, 8-µm thick transverse sections were obtained at 100 µm intervals throughout the muscle and then stained. The total number of GFP^+^ myofibres per transplanted muscle was recorded. Statistical significance was determined by the student *t*-test.

### Non-invasive bioluminescence imaging

Recipient mice were anaesthetized via isoflurane inhalation and imaged using a Xenogen IVIS 200 device. Briefly, the system contains a light-tight imaging chamber, a charge-coupled device camera and the appropriate computer system equipped with Living Image 3.2 software. Following intraperitoneal injection of 0.1 mmol/g body weight luciferin, images were acquired every 60 s for 15 min. In subsequent analysis, images containing peak luminescence values over this time period were used.

### RT-PCR

Total RNA was extracted with Trizol, and 0.5–1 µg were retrotranscribed using the Taqman reverse transcription kit (Applied Biosystems). Real time quantitative PCR was performed to analyse relative gene expression levels using SYBR Green Master mix (Applied Biosystems) following manufacturer indications. Relative expression values were normalized to the housekeeping gene GAPDH.

Primers sequences are as follow:

GAPDH:

Fw: 5′-CACCATCTTCCAGGAGCGAG-3′
Rev: 5′-CCTTCTCCATGGTGGTGAAGAC-3′

MyHC 3:

Fw: 5′-CAATAAACTGCGGGCAAAGAC-3′
Rev: 5′-CTTGCTCACTCCTCGCTTTCA-3′

MyHC 8:

Fw: 5′-GAACTTGAAGGAGAGGTCGA-3′
Rev: 5′-GAGCACATTCTTGCGGTCTT-3′

MCK:

Fwd: 5′-AGTCCTACACGGTCTTCAAGG-3′
Rev: 5′-AGGAAGTGGTCATCAATGAGC-3′

Follistatin:

Fwd: 5′-GCCTGCCACCTGAGAAAGG-3′
Rev : 5′-CGCCACACTGGATATCTTCACA-3′

### Statistical analysis

Data are presented as mean ± SEM. Comparisons between two groups were made using the Student's *t*-test assuming a two-tailed distribution, with significance being defined as *p < 0.05; **p < 0.01; ***p < 0.001. Comparisons between three or more groups were made using one-way Anova test, with Tukey's multiple comparisons post-test and with significance being defined as *p < 0.05; **p < 0.01; ***p < 0.001.

## Author contributions

CM made most of the experiments of the manuscripts with the help of SC and VS; MT performed transplantation experiments with cells from luciferase mice and bioluminescence analysis under the supervision of AS; AD sorted cells by FACS under the supervision of LB and GB; AS, KJM, DS and GM helped CM to set up FACS-mediated isolation of Sca1^pos^ cells from muscles. PLP designed the experiments of the manuscript, in collaboration with CM and AS, supervised them and wrote the manuscript. All the authors discussed, commented the results and read the manuscript.

## For more information

1. Sanford-Burnham Medical Research Institute:
2. http://www.sanfordburnham.org
3. ENDOSTEM:
4. http://www.endostem.eu/
5. Parent Project:
6. http://www.parentproject.org/
7. EPIGEN:
8. http://www.epigen.it/

## Notes

As a service to our authors and readers, this journal provides supporting information supplied by the authors. Such materials are peer reviewed and may be re-organized for online delivery, but are not copy-edited or typeset. Technical support issues arising from supporting information (other than missing files) should be addressed to the authors.
