# Supplementary material for: Fibroadipogenic progenitors mediate the ability of HDAC inhibitors to promote regeneration in dystrophic muscles of young, but not old Mdx mice
Source: EMBO Mol Med. 2013 Mar 18;5(4):626–39. doi: 10.1002/emmm.201202096 (PMC3628105; doi:10.1002/emmm.201202096)
Supplement: Supplementary file 1 [file emmm0005-0626-sd1.pdf]

EMBO Molecular Medicine

Fibroadipogenic progenitors mediate the ability of HDAC inhibitors to promote regeneration in dystrophic muscles of young, but not old mdx mice

Mozzetta C., Consalvi S., Saccone V., Tierney M., Diamantini A., Mitchel K.J., Marazzi G., Borsellino G., Battistini L., Sassoon D., Sacco A., Puri P.L.

Corresponding author: Pier Lorenzo Puri, SanfordBurnham Medical Research Institute

Review timeline:

Submission date:	01 October 2012
Editorial Decision:	17 November 2012
Additional Editorial Correspondence	21 November 2012
Revision received:	27 December 2013
Editorial Decision:	23 January 2013
Revision received:	02 February 2013
Accepted:	04 February 2013

Transaction Report:

(Note: With the exception of the correction of typographical or spelling errors that could be a source of ambiguity, letters and reports are not edited. The original formatting of letters and referee reports may not be reflected in this compilation.)

Editor: Roberto Buccione

1st Editorial Decision 17 November 2012

Thank you for the submission of your manuscript to EMBO Molecular Medicine.

We are very sorry for the delay in getting back to you with the Reviewers' evaluations. Unfortunately, in this case we experienced unusual difficulties in securing three appropriate reviewers in a timely manner. Since we cannot justify a further delay, we are sending the two consistent evaluations of Reviewers 1 and 3 at this time. We will forward Reviewer 2's report, as soon as we are able to obtain it.

You will see that while both Reviewers are generally supportive of your work and underline its considerable potential interest, they also both raise a number of specific concerns that prevent us from considering publication at this time.

Reviewer 1 feels that additional data is required to confirm that TSA is appropriately inhibiting HDACs in skeletal muscle. Indeed, the referee suggests that you complement the TSA data with MS-275.
Reviewer 1 also requests stronger in vivo data in fig. 6 to enhance the translational interest of the dataset (point 4).S/he also encourages experimentation to gain further mechanistic insight into how HDACs regulate fibroadipogenic progenitor-mediated myogenesis. While we acknowledge that this may not be feasible, we nonetheless strongly encourage developing the study as far as realistically possible in this direction.

Reviewer 3 is concerned that you do not conclusively demonstrate that the effect of TSA is specific for the fibroadipogenic progenitors and suggests an experimental approach to this effect. S/he also feels that immunofluorescence data showing enhanced differentiation should be supported by additional data. The Reviewer also raises a number of important criticisms about the statistics.

While publication of the paper cannot be considered at this stage, we would be pleased to consider a suitably revised submission in the future, provided, however, that the Reviewers' concerns are fully addressed with additional experimental data where appropriate.

As I noted, we still hope to receive the third review in the near future. If this report does arrive during the revision period and if it raises additional important issues that have to be addressed to support this study, these would also need to be taken into consideration in the revision.

Please note that it is EMBO Molecular Medicine policy to allow a single round of revision only and that, therefore, acceptance or rejection of the manuscript will depend on the completeness of your responses included in the next, final version of the manuscript.

As you know, EMBO Molecular Medicine has a "scooping protection" policy, whereby similar findings that are published by others during review or revision are not a criterion for rejection. However, I do ask you to get in touch with us after three months if you have not completed your revision, to update us on the status. Please also contact us as soon as possible if similar work is published elsewhere.

***** Reviewer's comments *****

Referee #1 (Comments on Novelty/Model System):

Mozzetta et al. have followed-up on their prior studies showing that the HDAC inhibitor, trichostatin A (TSA), increases the size and function of myofibers in dystrophin-deficient (MDX) mice by stimulating expression of the myostatin antagonist, follistatin. The current studies focus on further elucidating the mechanism of action of TSA in the model. As regeneration capacity of dystrophic muscle declines, there is progressive replacement of muscle tissue with fibrotic scars. Resident fibro-adipocyte progenitors (FAPs) appear to play an important role in governing the balance between muscle regeneration and scarring in the context of muscular dystrophy. Here, the authors show that FAPs from young mice stimulate muscle satellite cells to form myotubes/myofibers. This stimulatory effect is enhanced by TSA, both in cell culture and in vivo; TSA concomitantly suppresses the ability of FAPs to differentiate into adipocytes. In contrast, TSA was unable to block adipogenesis or enhance FAP-mediated myogenesis when FAPs were derived from old mice. Importantly, transplantation of FAPs from young mice into muscle of old MDX mice rescued the ability of TSA to increase myofiber size, suggesting therapeutic potential. Collectively, the authors provide fairly convincing evidence to support their interpretation that FAPs are critical mediators of satellite cell differentiation, and HDAC inhibition increases the myogenic potential of FAPs. Nonetheless, several issues dampen my enthusiasm for the manuscript in its current form.

Specific Points:

1. Several findings need to be assessed for statistical significance, for example Fig. 1C and Fig. 4A and 4C, since critical conclusions are made based on the data.
2. The authors need to confirm that TSA is appropriately inhibiting HDACs in skeletal muscle tissue by showing histone acetylation immunoblotting data. Yes, TSA is a potent HDAC inhibitor. However, it has an extremely short half-life in vivo. These results are particularly important since TSA is the only HDAC inhibitor used for the current studies. Why not also include MS-275, as was done in prior studies.
3. Given their prior findings, the authors should examine the impact of FAPs and TSA on follistatin expression.
4. For the in vivo studies shown in Fig. 6, the authors should quantify tissue fibrosis and also muscle mechanical properties. The latter results have significant relevance to the translational potential of the current findings.

5. A major limitation of the work is that the mechanism(s) by which TSA enhances FAP-mediated myogenesis is not addressed. What are the substrates and/or gene targets of HDACs that govern these effects?

Referee #1 (General Remarks):

Mozzetta et al. have followed-up on their prior studies showing that the HDAC inhibitor, trichostatin A (TSA), increases the size and function of myofibers in dystrophin-deficient (MDX) mice by stimulating expression of the myostatin antagonist, follistatin. The current studies focus on further elucidating the mechanism of action of TSA in the model. As regeneration capacity of dystrophic muscle declines, there is progressive replacement of muscle tissue with fibrotic scars. Resident fibro-adipocyte progenitors (FAPs) appear to play an important role in governing the balance between muscle regeneration and scarring in the context of muscular dystrophy. Here, the authors show that FAPs from young mice stimulate muscle satellite cells to form myotubes/myofibers. This stimulatory effect is enhanced by TSA, both in cell culture and in vivo; TSA concomitantly suppresses the ability of FAPs to differentiate into adipocytes. In contrast, TSA was unable to block adipogenesis or enhance FAP-mediated myogenesis when FAPs were derived from old mice. Importantly, transplantation of FAPs from young mice into muscle of old MDX mice rescued the ability of TSA to increase myofiber size, suggesting therapeutic potential. Collectively, the authors provide fairly convincing evidence to support their interpretation that FAPs are critical mediators of satellite cell differentiation, and HDAC inhibition increases the myogenic potential of FAPs. Nonetheless, several issues dampen my enthusiasm for the manuscript in its current form.

Specific Points:

1. Several findings need to be assessed for statistical significance, for example Fig. 1C and Fig. 4A and 4C, since critical conclusions are made based on the data.
2. The authors need to confirm that TSA is appropriately inhibiting HDACs in skeletal muscle tissue by showing histone acetylation immunoblotting data. Yes, TSA is a potent HDAC inhibitor. However, it has an extremely short half-life in vivo. These results are particularly important since TSA is the only HDAC inhibitor used for the current studies. Why not also include MS-275, as was done in prior studies.
3. Given their prior findings, the authors should examine the impact of FAPs and TSA on follistatin expression.
4. For the in vivo studies shown in Fig. 6, the authors should quantify tissue fibrosis and also muscle mechanical properties. The latter results have significant relevance to the translational potential of the current findings.
5. A major limitation of the work is that the mechanism(s) by which TSA enhances FAP-mediated myogenesis is not addressed. What are the substrates and/or gene targets of HDACs that govern these effects?

Referee #3 (General Remarks):

The manuscript by Mozzetta et al identifies fibroadipogenic progenitors (FAPs) as the cellular target of HDAC inhibitors (HDACi) in promoting muscle regeneration. Notably, the authors report that this FAP-mediated HDACi effect selectively influence the regeneration potential of satellite cells in mdx mice at early stages of disease. The authors ultimately conclude that "FAPs are key cellular determinants of disease progression in mdx mice and mediate a previously unappreciated stage-specific beneficial effect of HDACi in dystrophic muscles." The work certainly contains data of interest for the biology of skeletal muscle regeneration and for potential future therapeutics. The manuscript is well written and the experiments support the conclusions. However, some general and specific issues need to be addressed:
1) Introduction and results: other Sca1+ unconventional myogenic progenitors such as pericyte-derived mesoangioblasts need to be discussed. Moreover, how do the authors exclude that the HDACi effect is specific for FAPs and do not involve other myogenic progenitors? I would suggest

to perform an experiment (even an in vitro evidence would be sufficient) to define the specificity of the effect on FAP vs. other progenitors such as PICs and mesoangioblasts, for which mouse models and or markers are available for perspective isolation (PW1 and alkaline phosphatase, respectively; Mitchell KJ, Nat Cell Biol 2010; Dellavalle A et al., Nat Communications 2011).
2) Results, Fig. 1C: Please perform appropriate statistical analysis to support the conclusion (one-way ANOVA with post-test to compare groups).
3) Results: "Exposure to TSA did not substantially alter the FACS profiles in all the experimental conditions, as compared to untreated animals (data not shown)": Please provide this data as supplementary figure.
4) Results, Fig. 4E,F: Please perform appropriate statistical analysis.
5) Results, Fig.5B: The immunofluorescence in this and other experiments showing enhanced differentiation should be supported by additional evidence (i.e., qRT-PCR and/or western blots).
6) Results, "Figure 5D shows that FAPs from TSA-treated mdx mice enhanced at all time points the luciferase signals from co-transplanted MuSCs (Fig. 5D and E)": From the graph it appears that this is actually significant only at two time-points (4 and 6 days). Please clarify.
7) Results, Fig. 6A-C: This experiments is very interesting. However, I have some general concerns regarding GFP signal in skeletal muscle upon immunofuorescence staining (Jackson KA et al., Stem Cells. 2004;22(2):180-7) and I would appreciate to see the same result using anti-GFP immunohistochemistry (or by seeing the same result using Rosa26 mice as donors). Moreover, Fig. 6C shows statistical analysis of three groups using t-test: This should be done with ANOVA, since t-test is not appropriate to compare more than 2 groups.
8) Results, Fig 6E: It would be important to provide more evidence for this final experiment (e.g., H&E staining, Masson trichrome and morphometric analyses).
9) Discussion: It should be made clear that so far the human counterpart of Sca1 has not been identified yet and this is a major hurdle to translate this work in the human setting.
10) It is not always clear throughout the text the number of mice used in each experiment. Please specify at least in the figure legends.

Additional Editorial Correspondence 21 November 2012

We have now received the comments from Reviewer 2. As you will see S/he is quite positive and has no specific concerns for you to deal with.

I look forward to receiving your revised manuscript

***** Reviewer's comments *****

Reviewer #2 (Comments on Novelty/Model System):

This paper presents a number of interesting finding that contribute to explain the previously reported effects of TSA on muscular dystrophy mouse models. Given that this treatment has significant translation potential, this is a very appropriate topic for the journal's readership. The data appears convincing and of high quality, and they clearly establish the notion that TSA acts at least in part through enhancing the trophic effects that mesenchymal progenitors physiologically exert during regeneration, and preserving it from an age-associated decline. The inhibition of adipogenesis by TSA is less of a novel finding, but in the context it helps explain the observed effects of this drug.

Reviewer #2 (General Remarks):

I apologize for taking so long in reviewing this paper. This is particularly negligent as I actually reviewed a former version of this work for Cell Stem Cell. Despite going through two rounds of review and in the process addressing a number of my criticisms, that previous version contained some poorly substantiated claims related to an expansion of developmental potential in FAPs and

was rejected. As the "offending" claims have been completely removed from the current version, which is focused on the more solid findings that TSA modulates the trophic effects of FAPs on MuSC, I have no further criticism and would be happy to see this paper published as is.

1st Revision - authors' response 27 December 2013

We are pleased to see that all reviewers commented positively on our work and supported the publication, pending resolution of specific concerns.

We have worked to address the reviewer points, as outlined in the point-to-point reply below, and hope that you will find our answers satisfactory to proceed toward publication of this work in EMBO Molecular Medicine.

Point-to-point reply

While we were glad to learn that Referee # 2 did not express further criticism and would be happy to see the paper published as is.

Referee # 1

1) Several findings need to be assessed for statistical significance, for example Fig. 1C and Fig. 4A and 4C, since critical conclusions are made based on the data.

RE: We have introduced the statistical analysis (one-way ANOVA with post-test) of figures 1C, 4A and 4F (the review is probably referring to Fig. 4F instead of Fig. 4C) in the revised version of the manuscript.

2) The authors need to confirm that TSA is appropriately inhibiting HDACs in skeletal muscle tissue by showing histone acetylation immunoblotting data. Yes, TSA is a potent HDAC inhibitor. However, it has an extremely short half-life in vivo. These results are particularly important since TSA is the only HDAC inhibitor used for the current studies. Why not also include MS-275, as was done in prior studies.

RE: The ability of TSA to induce histone hyper acetylation in treated mdx mice has been already shown in our previous publication (Minetti et al. 2006). Likewise, we have previously shown that MS-275 promotes the same beneficial effects as TSA in mdx mice (Colussi et al. 2008). Therefore, we believe that such redundant information can be just provided by citing the appropriate literature.

3. Given their prior findings, the authors should examine the impact of FAPs and TSA on follistatin expression.

RE: We welcomed the reviewer suggestion, as we have included in the revised manuscript a new figure (Fig. 7) and paragraph (Follistatin is soluble mediator of functional interactions between FAPs and MuSCs from mdx mice exposed to HDACi) showing that FAPs display abundant expression of follistatin (about 10 fold higher than that detected in muscle satellite cells) and TSA up regulates follistatin levels in FAPs. We also show in the same figure that follistatin is an essential mediator of the functional interactions between FAPs and muscle satellite cells in basal conditions and after exposure to TSA – see below (point 5).

4. For the in vivo studies shown in Fig. 6, the authors should quantify tissue fibrosis and also muscle mechanical properties. The latter results have significant relevance to the translational potential of the current findings.

RE: We have quantified the fibrosis of old mdx mice transplanted with FAPs and treated with TSA, and observed no differences according to our prediction, based on the fact that fibrosis developed in old mdx mice cannot be reduced by such intervention, as also shown in Figure 3. Indeed, the scope of the experiment of FAP transplantation in old mdx mice shown in Figure 6 was aimed at demonstrating that young FAPs can restore the ability of TSA to promote an increase in CSA, as reflection of the restored activation of muscle satellite cell-mediated regeneration of old mdx muscles.

5. A major limitation of the work is that the mechanism(s) by which TSA enhances FAP-mediated myogenesis is not addressed. What are the substrates and/or gene targets of HDACs that govern these effects?

RE: In the revised version of the manuscript we show that TSA-induced follistatin in FAPs is one key mediator of functional interactions between FAPs and muscle satellite cells. Indeed, the new Fig. 7 shows that either follistatin down regulation by neutralizing antibodies against follistatin or shRNAi reduces FAP's ability to promote the myogenic potential of satellite cells in transwell co-culture experiments.

Referee #3

1) Introduction and results: other Sca1+ unconventional myogenic progenitors such as pericyte-derived mesoangioblasts need to be discussed. Moreover, how do the authors exclude that the HDACi effect is specific for FAPs and do not involve other myogenic progenitors? I would suggest to perform an experiment (even an in vitro evidence would be sufficient) to define the specificity of the effect on FAP vs. other progenitors such as PICs and mesoangioblasts, for which mouse models and or markers are available for perspective isolation (PW1 and alkaline phosphatase, respectively; Mitchell KJ, Nat Cell Biol 2010; Dellavalle A et al., Nat Communications 2011).

RE: The reviewer is correct and the point is well taken, as Sca1+ cells are extremely heterogeneous. Indeed, the cell types mentioned by the reviewers (PICs and pericyte-derived mesoangioblasts) are probably included, at least partially, in the Sca1+ cells. Thus, it is formally possible that these cell types might contribute to the observed ability of FAPs to promote satellite cell activity in co-culture. However, it is unlikely that PICs and pericyte-derived mesoangioblasts are the target for the ability of TSA to reduce fibroadipogensis, as these cells have not been shown to posses any fibro-adipogenic potential in previous studies (Mitchell KJ, Nat Cell Biol 2010; Dellavalle A et al., Nat Communications 2011). We certainly agree that this issue should be discussed more extensively (as we have done in the revised version of the manuscript); however, the experiment proposed by the reviewer is complicated by the fact that perspective isolation of these cells by PW1 and alkaline phosphatase has been shown in wild type mice, but mdx mice are not available for such purpose. At this stage it is fair to say that future studies should address the relative contribution of different sub-populations of Sca1+ cells to mediate functional interactions with satellite cells.

2) Results, Fig. 1C: Please perform appropriate statistical analysis to support the conclusion (one-way ANOVA with post-test to compare groups).

RE: one-way ANOVA with post-test have been included in the analysis of Fig. 1C, as well as Fig. 4A and F – see also below.

3) Results: "Exposure to TSA did not substantially alter the FACS profiles in all the experimental conditions, as compared to untreated animals (data not shown)": Please provide this data as supplementary figure.

RE: This data is now provided in the revised version of the manuscript, as supplementary Fig. 2

4) Results, Fig. 4E,F: Please perform appropriate statistical analysis.

RE: one-way ANOVA with post-test have been included in the analysis of Fig. 4A and F

5) Results, Fig.5B: The immunofluorescence in this and other experiments showing enhanced differentiation should be supported by additional evidence (i.e., qRT-PCR and/or western blots).

RE: In the revised version of the manuscript we show the increased levels of myogenic transcripts in satellite cells co-cultured with FAPs, with or without treatment with TSA. However, we have used the transwell co-culture setting, instead of standard co-culture (e.g. figure 5B, as suggested by the reviewer) as it provides a cleaner system.

6) Results, "Figure 5D shows that FAPs from TSA-treated mdx mice enhanced at all time points the luciferase signals from co-transplanted MuSCs (Fig. 5D and E)": From the graph it appears that this is actually significant only at two time-points (4 and 6 days). Please clarify.

RE: The reviewer is correct, and as the difference reported in figure5D is significant only at the time-points of 4 and 6 days. However, this does not compromise the overall result of the experiment, showing that FAPs from TSA-treated mdx mice enhanced the engraftment of co-transplanted MuSCs.

7) Results, Fig. 6A-C: This experiments is very interesting. However, I have some general concerns regarding GFP signal in skeletal muscle upon immunofluorescence staining (Jackson KA et al., Stem Cells. 2004;22(2):180-7) and I would appreciate to see the same result using anti-GFP immunohistochemistry (or by seeing the same result using Rosa26 mice as donors). Moreover, Fig. 6C shows statistical analysis of three groups using t-test: This should be done with ANOVA, since t-test is not appropriate to compare more than 2 groups.

RE: The GFP signal is indeed specific, as it derives from immunofluorescence with anti-GFP antibodies that did not generate any signal on the red channel (see laminin staining). Overall, spectral analysis at the confocal should be sufficient to rule out potential auto fluorescence artifacts. We do not agree that anti-GFP immunohistochemistry would help to address this specific issue, as the peroxidase signal might introduce additional caveats. To clarify this issue, we have included in the revised version of the manuscript the picture of muscles from untransplanted mice, showing no GFP signal.

8) Results, Fig 6E: It would be important to provide more evidence for this final experiment (e.g., H&E staining, Masson trichrome and morphometric analyses).

RE: See response to reviewer 1 point 4

9) Discussion: It should be made clear that so far the human counterpart of Sca1 has not been identified yet and this is a major hurdle to translate this work in the human setting.

RE: We have introduced this point in the discussion of the revised manuscript.

10) It is not always clear throughout the text the number of mice used in each experiment. Please specify at least in the figure legends.

RE: We introduced the number of mice used in each experiment in the figure legend of the revised manuscript.

2nd Editorial Decision 23 January 2013

Thank you for the submission of your revised manuscript "Fibroadipogenic progenitors mediate the ability of HDAC inhibitors to promote regeneration in dystrophic muscles of young, but not old mdx mice" to EMBO Molecular Medicine. We have now received the enclosed reports from the referees whom we asked to re-assess it. You will be glad to see that the reviewers are now supportive and we can proceed with official acceptance of your manuscript pending the following minor changes:

- Your manuscript is missing the "The Paper Explained section". EMBO Molecular Medicine articles are accompanied by a summary of the articles to emphasize the major findings in the paper and their medical implications for the non-specialist reader. Please provide a draft summary of your article highlighting
- the medical issue you are addressing,
- the results obtained and
- their clinical impact.
This may be edited to ensure that readers understand the significance and context of the research. Please refer to any of our published articles for an example.

- There is space at the end of each article ("For More Information") to list relevant web links for further consultation by our readers. Could you identify some relevant ones and provide such information as well? Some examples are patient associations, relevant databases, OMIM/proteins/genes links, author's websites, etc...

Please submit your revised manuscript within two weeks. I look forward to seeing a revised form of your manuscript as soon as possible.

***** Reviewer's comments *****

Referee #1 (General Remarks):

The authors have performed additional experiments and analyses and the manuscript is improved. I do disagree with their assertion that prior demonstration of histone acetylation in TSA treated animals (2006) is sufficient. Undoubtedly they are using a new lot of TSA that has been newly formulated and delivered by someone other than the investigator who performed the studies in 2006. Any acetylation data from the current study would be useful.

Referee #3 (General Remarks):

I am happy with the authors replies to my comments. The statistical analysis now appears to be properly done and overall the manuscript is improved. It also provides new and interesting data (e.g., figure 7).

2nd Revision - authors' response 02 February 2013

We are submitting the final version of the manuscript entitled “Fibroadipogenic progenitors mediate the ability of HDAC inhibitors to promote regeneration in dystrophic muscles of young, but not old mdx mice”, which has been accepted for publication. We have followed the instruction of the editor in the acceptance letter, and therefore "The paper explained" and "For more information" sections have been uploaded. Moreover, we have uploaded the updated version of the supplementary information PDF starting with a table of contents, as indicated by the editor
