# Supplementary material for: Fibroadipogenic progenitors mediate the ability of HDAC inhibitors to promote regeneration in dystrophic muscles of young, but not old Mdx mice
Source: EMBO Mol Med. 2013 Mar 18;5(4):626–39. doi: 10.1002/emmm.201202096 (PMC3628105; doi:10.1002/emmm.201202096)
Supplement: Supplementary file 2 [file emmm0005-0626-sd2.pdf]

Supportive Information Table of Contents:

-Supportive Information Figure 1
-Supportive Information Figure legend 1
-Supportive Information Figure 2
-Supportive Information Figure legend 2
-Supportive Information Figure 3
-Supportive Information Figure legend 3
-Supportive Information Figure 4
-Supportive Information Figure legend 4

Supportive information Figure 1

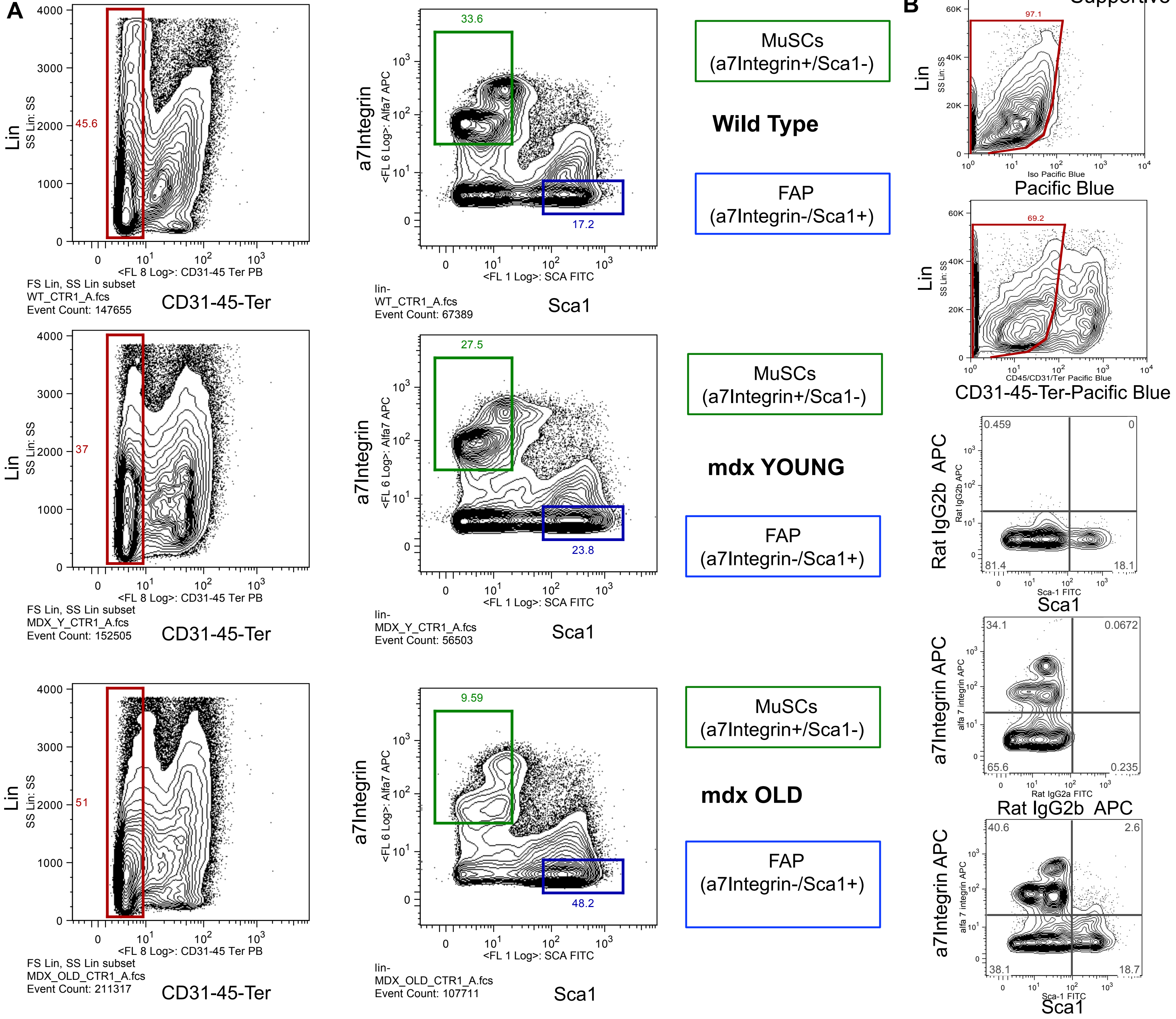

Supporting information Figure 1 - FACS mediated isolation of MuSCs and FAPs from wild type and mdx mice.
(A) Whole mononucleated cells from hindlimb skeletal muscles of wild type (upper panels) or young (1.5 month old; middle panels) and old (1 year old; bottom panels) mdx mice were selected based on morphological parameters, propidium iodide negativity and subjected to FACS, as indicated in the scheme. CD45+/CD31+/Ter119+ cells were negatively selected to exclude macrophages, hematopoietic and endothelial cells. Lineage negative cells (Ter119-/CD45-/CD31- cells; red box) were then separated on the basis of Sca-1 and α7-integrin relative expression. Muscle satellite cells (MuSCs) were isolated as α7-integrin+/Sca-1- cells (green box). Fibro-adipogenic progenitors (FAPs) were isolated as α7-integrin-/Sca-1+ cells (blue box). **(B)** FACS plots showing the fluorescence minus one (FMO) controls of antibodies used in (A).

Supportive Information Figure 2

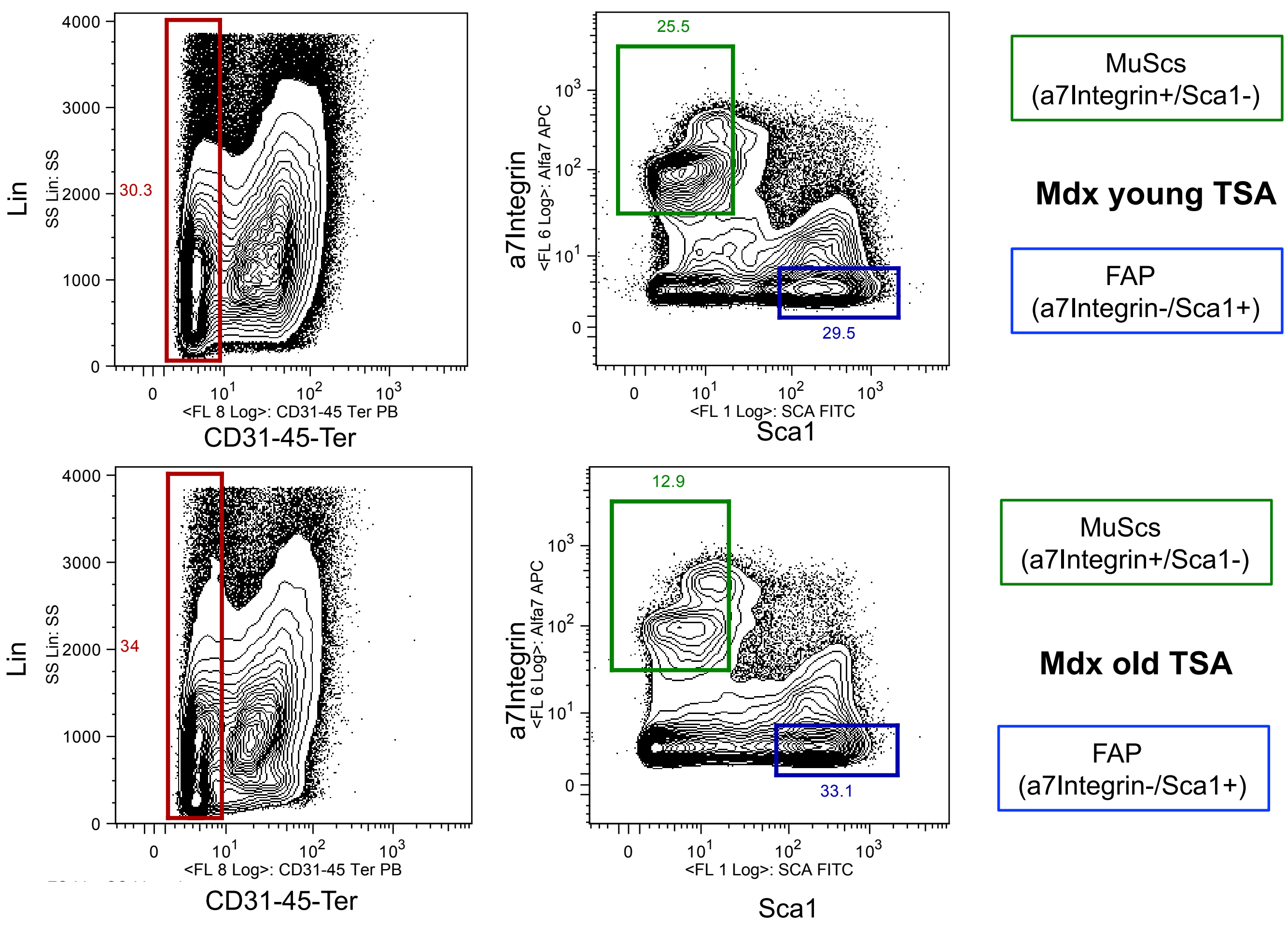

Supporting information Figure 2 - FACS mediated isolation of MuSCs and FAPs from TSA-treated mdx mice.

(A) Whole mononucleated cells from hindlimb skeletal muscles of young (1.5 month old; top panels) and old (1 year old; bottom panels) mdx mice treated with TSA were selected based on morphological parameters, propidium iodide negativity and subjected to FACS, as indicated in the scheme. CD45+/CD31+/Ter119+ cells were negatively selected to exclude macrophages, hematopoietic and endothelial cells. Lineage negative cells (Ter119-/CD45-/CD31- cells; red box) were then separated on the basis of Sca-1 and α7-integrin relative expression. Muscle satellite cells (MuSCs) were isolated as α7-integrin+/Sca-1- cells (green box). Fibro-adipogenic progenitors (FAPs) were isolated as α7-integrin-/Sca-1+ cells (blue box).

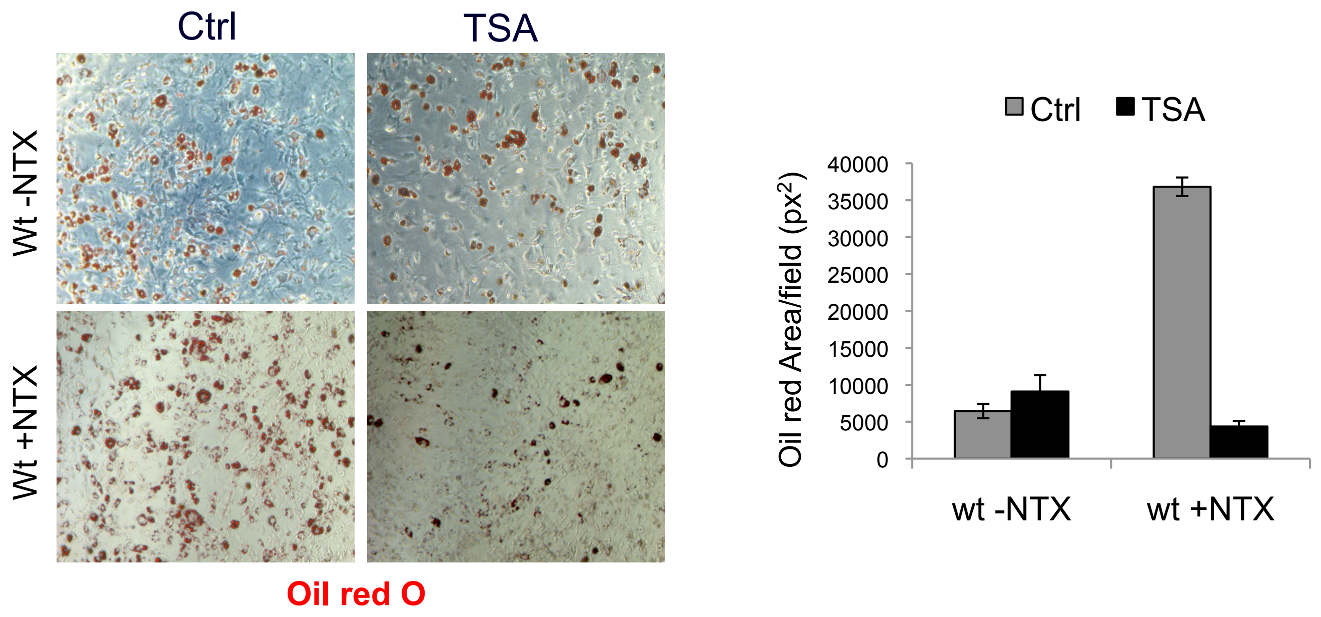

Ctrl
TSA
Wt -NTX
Wt +NTX
Oil red O
Ctrl
TSA
Oil red Area/field (px2)
40000
35000
30000
25000
20000
15000
10000
5000
0
wt -NTX
wt +NTX

Supportive information Figure 3 - HDACi repress the adipogenic potential of FAPs cells isolated from wild-type injured muscles.

Left Panel: Right hindlimb skeletal muscles of wild type mice were injured with notexin (NTX) and contro-laterals were left uninjured. After 10 days of treatment with vehicle (Ctrl) or TSA, FAPs where isolated by FACS and cultured in proliferating medium for 7 days, induced to differentiate in adipogenic differentiation medium (AdpDM) for 6 days and then stained by Oil Red O to assess adipogenic differentiation. Right Panel: Graph showing the quantification of Oil Red O areas (pixel^2) per field in the same conditions as in (A). Data are represented as average ± SEM.

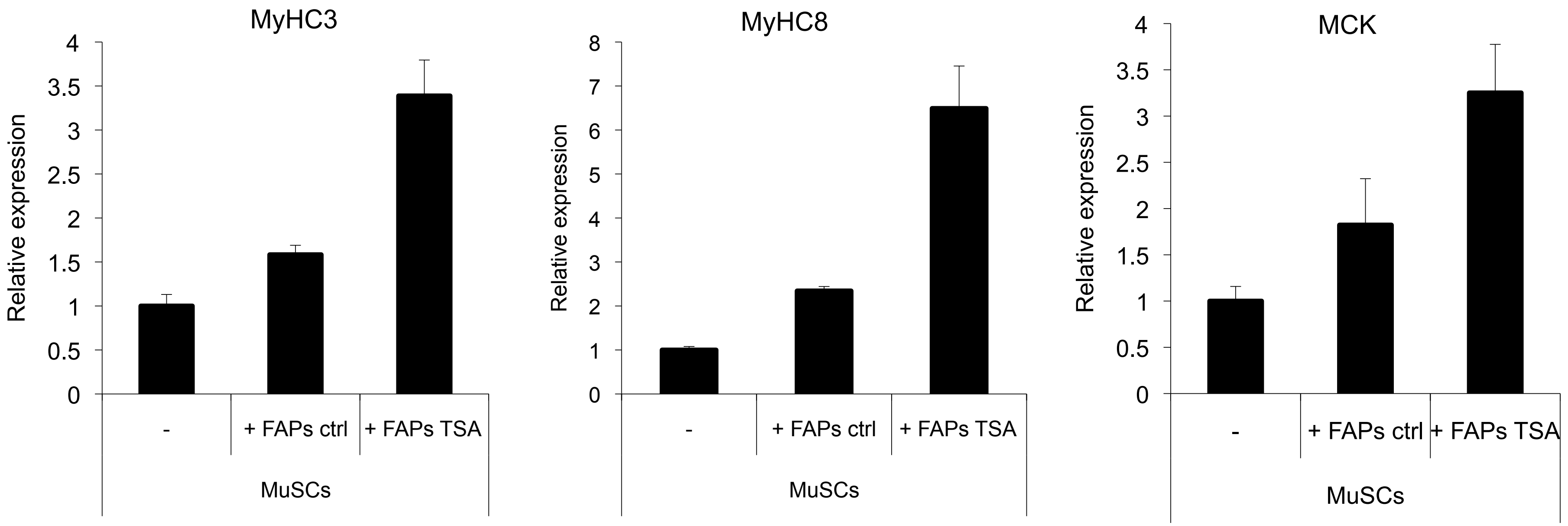
MyHC3
Relative expression
4
3.5
3
2.5
2
1.5
1
0.5
0
-
+ FAPs ctrl
+ FAPs TSA
MuSCs
MyHC8
Relative expression
8
7
6
5
4
3
2
1
0
-
+ FAPs ctrl
+ FAPs TSA
MuSCs
MCK
Relative expression
4
3.5
3
2.5
2
1.5
1
0.5
0
-
+ FAPs ctrl
+ FAPs TSA
MuSCs

Supportive information Figure 4 – HDACi-treated FAPs promote expression of muscle differentiation markers in co-cultured MuSCs.

FACS-isolated MuSCs from young MDX mice were co-cultured in transwell with FAPs isolated from young MDX mice (ctrl or TSA treated for 15 days). After 7 days of transwell co-culture the myogenic differentiation of MuSCs was assessed by qRT-PCR analyzing RNA levels of MyHC3, MyHC8 and MCK.
